# A Comprehensive Review on the Diagnosis of Knee Injury by Deep Learning-Based Magnetic Resonance Imaging

**DOI:** 10.7759/cureus.45730

**Published:** 2023-09-21

**Authors:** Neha D Shetty, Rajasbala Dhande, Bhavik S Unadkat, Pratapsingh Parihar

**Affiliations:** 1 Department of Radiodiagnosis, Datta Meghe Institute of Higher Education and Research, Wardha, IND

**Keywords:** meniscus tear, ligamentous knee injury, persistent knee pain, magnetic resonance imaging, deep-learning

## Abstract

The continual improvement in the field of medical diagnosis has led to the monopoly of using deep learning (DL)-based magnetic resonance imaging (MRI) for the diagnosis of knee injury related to meniscal injury, ligament injury including the cruciate ligaments, collateral ligaments and medial patella-femoral ligament, and cartilage injury. The present systematic review was done by PubMed and Directory of Open Access Journals (DOAJ), wherein we finalised 24 studies conducted on the accuracy of DL MRI studies for knee injury identification. The studies showed an accuracy of 72.5% to 100% indicating that DL MRI holds an equivalent performance as humans in decision-making and management of knee injuries. This further opens up future exploration for improving MRI-based diagnosis keeping in mind the limitations of verification bias and data imbalance in ground truth subjectivity.

## Introduction and background

The knee joint, one of the large complex joints, has been the topic of most discussions in view of the injuries to various anatomical structures - ligaments (anterior cruciate ligament, posterior cruciate ligament, medial collateral ligament and lateral collateral ligament), meniscus (medial or lateral) and cartilage [1,2]. Knee injuries are reported to affect nearly 244,000 people annually, according to an analysis that included National Health Fund (NHF) data from 2016-2019 [1]. The prevalence of knee pain was reported as 21.4% [3]. In an Indian study, including 517 patients who underwent primary anterior cruciate ligament reconstruction (ACLR), 70% had a meniscal injury and 50% had chondral damage [4]. Acute knee injury is generally caused because of direct trauma, or due to excess tension, sudden twists, collisions, awkward movements, falls, excessive force, and overuse of joints [5]. Shoe wear, training surface conditions, and training regimen are the extrinsic factors, whereas ligamentous laxity, muscle weakness, reduced muscle flexibility, and foot shape are the intrinsic factors [6]. The possible effects of knee injuries include tendinopathies and structural muscle injuries of the lower limb [7,8]. Early detection of ruptured ligament, meniscal tears, as well as cartilage lesions and consequent treatment, are necessary for management, which can also delay the onset of knee osteoarthritis following a knee injury [9].

For the diagnosis, arthroscopy remains the gold-standard modality. However, its use over time has been restricted and overpowered by newer non-invasive investigations like magnetic resonance imaging (MRI) [10]. However, diagnosis of knee injury by MRI can be difficult, with clinicians' experience being crucial in image interpretation and the best management of knee injuries is sometimes hampered by limitations of the human mind in interpreting the imaging reports by factors like a distraction, workload, subjectivity, image quality and knowledge [11]. Furthermore, clinical-diagnostic differences between orthopaedic surgeons and non-musculoskeletal radiologists are frequently seen in day-to-day clinical practice [12]. Considering the rising cases of knee injuries and sports injuries and the above-mentioned factors, the use of artificial intelligence (AI) has become rampant in medical practice, whereby certain diagnostic algorithms are created based on digital imaging data collection and interpolation. AI involves a technique that makes it possible for computers to complement human intelligence. The growth of AI is specifically being driven by deep learning (DL), which falls under the category of machine learning (ML) algorithms. There have been several documented uses of DL in image interpretation, including classifying skin carcinoma, detecting lung nodules, mammography cancer, and diabetic retinopathy. AI-powered technologies are anticipated to change the medical field as they increase the diagnostic accuracy of several diagnostic and therapeutic techniques [13]. A number of early DL investigations have shown improved performance over conventional ML processes and are even found to be superior to radiologists in the diagnosis of knee injuries [14]. The limitations of prior conducted similar systematic reviews were that they included other knee injuries like bony fractures [15] or failed to address the limitation and strengths of various AI in deciding the performance of the system [16]. Taking into account the emerging AI technology as well as increasing research in similar fields, in this systematic review we conducted a comprehensive analysis of the literature, encompassing all DL-based methodologies which are employed in knee injury diagnosis. The present systematic review aimed to identify the latest studies that evaluated the role of DL in MRI-based knee injury diagnosis. The main emphasis was laid on research that assessed knee ligamentous tear, meniscal tears or cartilaginous lesions.

## Review

Methods

Literature Search

**Figure 1:**
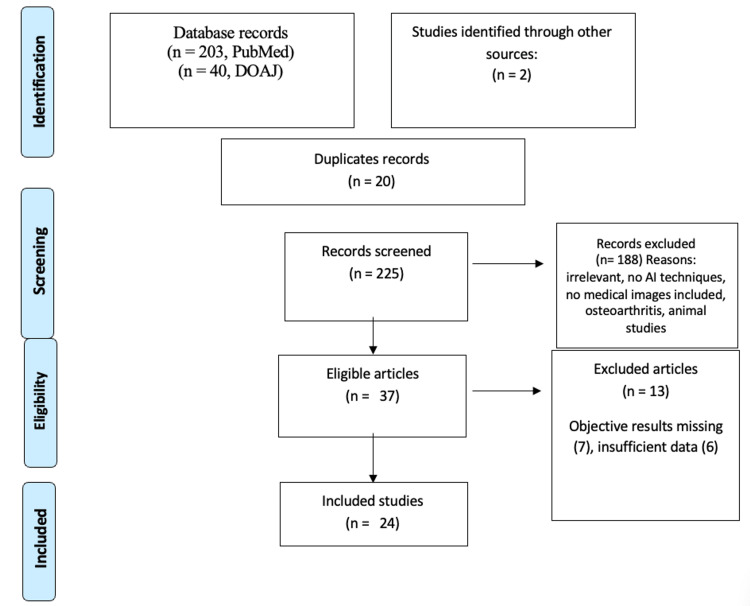
Preferred Reporting Items for Systematic Reviews and Meta-Analyses (PRISMA) Flowchart. DOAJ: Directory of Open Access Journals

We conducted a thorough search of studies as per the Preferred Reporting Items for Systematic Reviews and Meta-Analyses (PRISMA) guidelines, wherein two primary databases, PubMed and Directory of Open Access Journals (DOAJ), were reviewed by two primary authors and the data was extracted by the third author. An electronic data search was conducted on the databases of PubMed and DOAJ over a period of the last 10 years of published studies with specified dates from January 2013 till January 2023. For the database search, Medical Subject Headings (MeSH) keywords were used as per the PubMed dictionary separated by Boolean expression: "knee"[MeSH Terms] OR "knee joint"[MeSH Terms] AND "magnetic resonance imaging"[MeSH Terms] AND "machine learning"[MeSH Terms] in “All fields” as tag terms. The text words used in DOAJ were the same as MeSH terms used in PubMed without any additional filters. The articles were found to be eligible based on the title and the abstract. The full text of the articles was studied only after verification of their abstract and the title and the inclusion criteria (Figure 1).

Inclusion Criteria

Only full texts and papers were chosen for the study. English articles were included which were published over the last 10 years from January 2013 till January 2023. Studies that diagnosed knee injury based on the MRI deep learning AI-based algorithms and studies that mentioned the accuracy of the performance of AI algorithms were included.

*Exclusion Criteria*

Exclusion criteria were articles published before January 2013, articles dealing with injuries other than knee injuries, studies published on animals, studies other than original articles, i.e. review articles, systematic research or meta-analysis, studies written in a language other than English, and editorial commentaries and book chapters. 

Data Extraction

After downloading all the studies, the data was extracted and put in a table in Microsoft Spreadsheet. The information extracted from each article included the first name of the author, the year of publication, the description of data, the learning algorithm used, the sample size, the type of injury, and the accuracy of the DL model for diagnosis of a knee injury. 

Assessment of Risk of Bias

The assessment of the risk of bias was done by using the ROBINS-I tool which consists of seven parameters that include selection bias, measurement bias, attrition bias, confounding bias, performance bias, and outcome reporting bias. A total score categorized as low, moderate, and high was classified, wherein the presence of none of these confounding factors or one of these confounding factors led to the classification of low and two or more up to five led to the classification of moderate and five or more up to seven led to the classification of high risk of bias as for the eligible studies is shown in Table 1 [17].

**Table 1:** Risk of Bias Assessment L: Low, M: Moderate

Name of the author	Confounding bias	Selection bias	Measurement on interventions bias	Intended interventions (performance bias)	Attrition bias	Measurement bias	Outcome reporting bias	Overall Risk of bias
Li J et al, 2022 [18]	-	+	+	+	-	-	Y	M
Li Z et al, 2021 [19]	-	-	-	-	-	-	-	L
Awan et al, 2021 [20]	-	+	-	-	+	-	-	M
Jeon et al, 2021 [21]	-	+	-	-	+	-	-	M
Rizk et al, 2021 [22]	-	-	-	-	-	-	-	L
Dai et al, 2021 [23]	+	-	+	+	-	-	+	M
Astuto et al, 2021 [24]	-	+	-	-	+	-	-	M
Fritz et al, 2020 [25]	+	-	-	+	+	-	+	M
Namiri et al, 2020 [26]	-	-	-	-	-	-	-	L
Zhang et al, 2020 [27]	-	-	+	+	-	-	+	M
Germann et al, 2020 [28]	+	-	-	+	+	-	+	M
Azcona et al, 2020 [29]	-	-	-	-	-	+	-	L
Chang et al, 2019 [30]	-	+	-	-	+	-	-	M
Liu et al, 2019 [31]	-	+	-	-	+	-	-	M
Couteaux et al, 2019 [32]	-	-	+	+	-	-	+	M
Pedoia et al, 2019 [33]	-	-	-	-	-	-	-	L
Roblot et al, 2019 [34]	-	+	+	-	-	-	+	M
Bien N et al, 2018 [13]	+	-	-	+	+	-	+	M
Liu et al, 2018 [35]	+	-	+	-	-	-	+	M
Štajduhar et al, 2017 [36]	-	+	-	-	+	-	-	M
Mazlan et al, 2017 [37]	+	-	-	-	+	-	-	M
Zarandi et al, 2016 [38]	-	-	-	-	-	-	-	L
Fu et al, 2013 [39]	+	-	-	-	-	-	-	L
Abdullah et al, 2013 [40]	-	-	+	+	-	-	+	M

Results

Search Results

Based on our search, we identified 203 articles in PubMed, 40 articles in DOAJ, and two articles from other references of the selected articles. Among them, there were 20 duplicate articles, which were excluded, and 225 articles were screened by two primary authors. Among them, 188 were excluded based on reasons that fell into the exclusion criteria and 37 full-text articles were downloaded. They were thoroughly screened, and 24 out of them were selected, and 13 were excluded since they did not have sufficient data to qualify for the systematic review. Finally, 24 studies were included in the systematic review, the characteristics of which are shown in Table 2.

**Table 2:** Study Characteristics DL: Deep Learning; ANN: Artificial Neural Networks; BP-ANN: Back Propagation ANN; CNN: Convolutional Neural Network; DCNN: Deep CNN; GIST: Generalized Search Tree; HOG: Histogram of Oriented Gradient; IT2FCM: Interval Type-2 Fuzzy C-Means; K-NN: K-Nearest Neighbor; PNN: Perceptron Neural Network; R-CNN: Region-Based CNN; SVM: Support Vector Machine; ACL: Anterior Cruciate Ligament

S.no.	Author	N	Patient injury	DL model (AI model used)	MRI used (Tesla)	Accuracy of DL	
1.	Li J et al, 2022 [18]	200	Meniscus tear	Mask R-CNN	1.5 T and 3.0 T	Healthy: 87.50% Torn: 86.96% Degenerated meniscus: 84.78%	
2.	Li Z et al, 2021 [19]	30	ACL	CNN	2.0	92.17%	
3.	Awan et al, 2021 [20]	917 images	ACL tear	CNN	1.5 T	Partial ACL tear: 0.97; full ACL tear: 0.99	
4	Jeon et al, 2021 [21]	2540	ACL tear	3D CNN	3 T & 1.5 T	0.98	
5	Rizk et al, 2021 [22]	7903	Meniscus tear	3D CNN	1-3 T	Medial = 0.93, Lateral = 0.84	
6	Dai et al, 2021 [23]	1714	ACL tear, Meniscus tear	TransMed	3 T & 1.5 T	94.9%/0.98, 85.3%/0.95	
7	Astuto et al, 2021 [24]	294	ACL tear—Meniscus tear—Cartilage lesion	3D CNN	3T	from 0.83 to 0.93	
8	Fritz et al, 2020 [25]	100	Meniscus tear	DCNN	1.5 T (64%)–3 T (36%)	Medial = (86%/0.88), Lateral = (84%/0.78), Overall = (0.96)	
9	Namiri et al, 2020 [26]	224	ACL tear	CNN	3T	3Dmodel = (89%/sensitivity of 89% and specificity of 88%), 2Dmodel = (92%/sensitivity of 93% and specificity of 90%)	
10	Zhang et al, 2020 [27]	408	ACL tear	CNN	1.5 T (74%)–3 T (26%)	95.7%	
11	Germann et al, 2020 [28]	512	ACL tear	DCNN	1.5 T–3 T	0.94	
12	Azcona et al, 2020 [29]	-	ACL tear, Meniscus tear	CNN	1.5 T, 3 T	0.96, 0.91	
13	Chang et al, 2019 [30]	260	ACL tear	CNN	1.5 T–3 T	96.7%/0.97	
14	Liu et al, 2019 [31]	175	ACL tear	CNN	N/A	0.98	
15	Couteaux et al, 2019 [32]	1128 images	Meniscus tear	CNN	N/A	0.90	
16	Pedoia et al, 2019 [33]	302	Meniscus tear	2D U-Net, CNN	3T	Sensitivity of 89.81% and specificity of 81.98%	
17	Roblot et al, 2019 [34]	1123 images	Meniscus tear	CNN	N/A	72.5%/0.85	
18	Bien N et al, 2018 [13]	Training set: 1,088 patients Tuning set: 111 patients Validation set: 113 patients	ACL tear—Meniscus tear— Abnormalities	CNN	3 T (56.6%)–1.5 T (43.4%)	86.7%/0.97–72.5%/0.85– 0.94	
19	Liu et al, 2018 [35]	175	Cartilage lesion	CNN	3T	0.92	
20	Štajduhar et al, 2017 [36]	969 images	ACL tear	HOG, GIST, RF	1.5T	(Injury detection problem, complete rupture) = (0.89, 0.94), (0.88, 0.94), (0.889, 0.91), (0.88, 0.90) respectively with the models	
21	Mazlan et al, 2017 [37]	300 images	ACL tear	SVM	N/A	100%	
22	Zarandi et al, 2016 [38]	28	Meniscus tear	IT2FCM, PNN	N/A	90%,78%	
23	Fu et al, 2013 [39]	166 images	Meniscus tear	SVM	N/A	SVM model: 0.73 SFFS + SVM: 0.91	
24	Abdullah et al, 2013 [40]	90 images	ACL tear	BP ANN, K-NN	N/A	BP ANN: 94.44% k-NN: 87.83%	

Study characteristics

The countries where the included studies were conducted were France [22,32,34], Switzerland [25,28], Ireland [29], USA [13,20,24,26,30,31,33,35], Turkey [36] and Asia [18,19,21,23,27,37-40]. The study period in those studies varied from five years to 18 years. 

Study outcomes

Anterior cruciate ligament (ACL) injuries were present in 16 studies [13,18,20,21,23,24,26-31,36,37,40], meniscus injuries in 12 studies [13,19,22-25,29,32-34,38-40], and cartilage lesion in two studies [24,35]. 

Accuracy of the artificial intelligence model

The overall accuracy of the AI model was 72.5% to 100% for knee injuries.

Risk of bias

In seven studies, the risk of bias was low [19,22,26,29,33,38,39] and in the remaining, i.e. 17 studies, the moderate risk was present [13,18,20,21,23-25,27,28,30-32,34-37,40].

Discussion

The present systematic review holds significance as it summarized all the studies that used deep learning MRI models for the diagnosis of knee injuries. Deep learning-based MRI is basically a part of artificial intelligence which is expanded in various domains in the entire world [41]. Performance and accuracy as seen among the studies ranged from 72.5% to 100% which is effective enough for expanding the medical experts’ knowledge and diagnostic skills for knee injuries. Our findings were in line with a previous systematic review conducted by Siouras et al. [41], who also quoted diagnostic accuracy of 72.5-100%, but the review was conducted on 22 studies. Another systematic review was conducted by Kunze et al. [16] including 11 studies, among which five evaluated ACL tears, five assessed meniscal tears, and one study assessed both where the area under the curve (AUC) for detecting ACL tear was in the range of 0.895 to 0.980 and for meniscus tear was 0.847 to 0.910. The use of deep learning has come frequently into practice since there is no gold standard scoring system to diagnose knee injuries. Artificial intelligence complements the human mind in a machine-operated way where the probability of diagnosing the injured knee becomes higher. This has been mainly based on the data augmentation and data acquiring for ACL, meniscal and cartilage injuries. Moreover, this has become possible through various image transformations which include shifting, and flipping rotations, thereby expanding the dataset and improving the performance of DL-based learning and diagnosis [41]. We noticed that studies reported a difference in the performance of deep learning MRI-based diagnosis accuracy and this variability in the performance of different studies might be because of the region of interest that may appear slightly different within an image and because of the different ratios and sizes. The lowest accuracy of 72.5% was reported by Roblot et al. [34] and Bien et al. [13], which was primarily on the diagnosis of meniscus tears using convolutional neural network, while the highest accuracy was reported by Mazlan et al. [37] of 100%, which was specifically on ACL tears with the AI model of support vector machine (SVM) being used which gives high data gap between injury data (actual data) and non-injury data. This shows that variability in performance can be due to data imbalance whereby patients have different grades of knee injuries, and the application of a uniform algorithm may not be as effective. So, multiple datasets are needed whereby deep learning can be effectively improved by expanding the MRI protocol thereby allowing it to perform in equivalence to the human mind - stressing the role of different AI models to be used and evaluation of the wide type of knee injuries to improve on the accuracy. The present systematic review holds strength in this regard since it covers many regions allowing for data pooling of three categories of knee injuries - thereby representing the multiset of data with the elimination of the bias and determining the effective performance of deep learning-based MRI.

Limitations

The present study was a systematic review, but it has certain limitations. Firstly, the meta-analysis was not done. Secondly, studies do not assess the combined use of machine learning and human learning. Thirdly, the gold standard diagnostic arthroscopy was not done in all the studies, which may have restricted the clinical applicability of the findings of the present study. In view of these limitations, future studies are recommended to expand the datasets and test the accuracy of machine deep learning-based MRI for the detection of knee injuries, especially meniscal, ACL, and cartilage injuries, and compare them with the gold standard non-invasive tests so that their applicability can be put to use soon. This shall help in the future to cover up for the workload which is expanding by leaps and bounds, whereby radiological imaging data has been expanding, but the number of radiologists is not increasing proportionally. The decision-making process is also hampered on this account. Thus AI systems can be a boon to humans. This is also essential because medical imaging is very sensitive in nature whereby quality and resolution demand high performance with minimal differences in diagnosing specific knee injuries. Training and specialization of the human eye and mind may require long experience before one may come to terms with machine-based learning [12-14].

Future directions

The findings of the present study show the different applications of deep learning MRI used till now, and there is still so much scope to fully exploit the full potential of this data where newer algorithms can be laid down by individual hospitals for making trustworthy detection systems for knee injuries. This demands further AI expandability and a collaboration of medical and IT fields to reach precision medicine. 

## Conclusions

Deep-based learning methods have come a long way, showing performance accuracy of more than 75%, leading to significant use in clinical employment. However, there are so many algorithms to be laid down, and individual datasets need to be expanded by multiregional studies whereby deep-based MRI detection can become a norm in every hospital, thereby retaining the high-performance standards and yielding faster diagnosis and management.

## Appendices

**Table 3:** List of articles searched from PubMed database

PMID	Title	Authors	Citation	First Author	Journal/Book	Publication Year	Create Date	PMCID	NIHMS ID	DOI
34804143	Emergence of Deep Learning in Knee Osteoarthritis Diagnosis	Yeoh PSQ, Lai KW, Goh SL, Hasikin K, Hum YC, Tee YK, Dhanalakshmi S.	Comput Intell Neurosci. 2021 Nov 10;2021:4931437. doi: 10.1155/2021/4931437. eCollection 2021.	Yeoh PSQ	Comput Intell Neurosci	2021	2021/11/22	PMC8598325		10.1155/2021/4931437
35204625	Knee Injury Detection Using Deep Learning on MRI Studies: A Systematic Review	Siouras A, Moustakidis S, Giannakidis A, Chalatsis G, Liampas I, Vlychou M, Hantes M, Tasoulis S, Tsaopoulos D.	Diagnostics (Basel). 2022 Feb 19;12(2):537. doi: 10.3390/diagnostics12020537.	Siouras A	Diagnostics (Basel)	2022	2022/02/25	PMC8871256		10.3390/diagnostics12020537
32722605	A Comparative Systematic Literature Review on Knee Bone Reports from MRI, X-rays and CT Scans Using Deep Learning and Machine Learning Methodologies	Khalid H, Hussain M, Al Ghamdi MA, Khalid T, Khalid K, Khan MA, Fatima K, Masood K, Almotiri SH, Farooq MS, Ahmed A.	Diagnostics (Basel). 2020 Jul 26;10(8):518. doi: 10.3390/diagnostics10080518.	Khalid H	Diagnostics (Basel)	2020	2020/07/30	PMC7460189		10.3390/diagnostics10080518
35184211	Inferring pediatric knee skeletal maturity from MRI using deep learning	Zech JR, Carotenuto G, Jaramillo D.	Skeletal Radiol. 2022 Aug;51(8):1671-1677. doi: 10.1007/s00256-022-04010-y. Epub 2022 Feb 20.	Zech JR	Skeletal Radiol	2022	2022/02/20			10.1007/s00256-022-04010-y
35529263	Deep Learning-Based Multimodal 3 T MRI for the Diagnosis of Knee Osteoarthritis	Hu Y, Tang J, Zhao S, Li Y.	Comput Math Methods Med. 2022 Apr 29;2022:7643487. doi: 10.1155/2022/7643487. eCollection 2022.	Hu Y	Comput Math Methods Med	2022	2022/05/09	PMC9076302		10.1155/2022/7643487
36648347	Deep Learning Reconstruction Enables Prospectively Accelerated Clinical Knee MRI	Johnson PM, Lin DJ, Zbontar J, Zitnick CL, Sriram A, Muckley M, Babb JS, Kline M, Ciavarra G, Alaia E, Samim M, Walter WR, Calderon L, Pock T, Sodickson DK, Recht MP, Knoll F.	Radiology. 2023 Apr;307(2):e220425. doi: 10.1148/radiol.220425. Epub 2023 Jan 17.	Johnson PM	Radiology	2023	2023/01/17	PMC10102623		10.1148/radiol.220425
35776434	Deep Learning-Enhanced Parallel Imaging and Simultaneous Multislice Acceleration Reconstruction in Knee MRI	Kim M, Lee SM, Park C, Lee D, Kim KS, Jeong HS, Kim S, Choi MH, Nickel D.	Invest Radiol. 2022 Dec 1;57(12):826-833. doi: 10.1097/RLI.0000000000000900. Epub 2022 Jul 1.	Kim M	Invest Radiol	2022	2022/07/01			10.1097/RLI.0000000000000900
35262842	Automatic detection and classification of knee osteoarthritis using deep learning approach	Abdullah SS, Rajasekaran MP.	Radiol Med. 2022 Apr;127(4):398-406. doi: 10.1007/s11547-022-01476-7. Epub 2022 Mar 9.	Abdullah SS	Radiol Med	2022	2022/03/09			10.1007/s11547-022-01476-7
34142088	Automatic Deep Learning-assisted Detection and Grading of Abnormalities in Knee MRI Studies	Astuto B, Flament I, K Namiri N, Shah R, Bharadwaj U, M Link T, D Bucknor M, Pedoia V, Majumdar S.	Radiol Artif Intell. 2021 Jan 20;3(3):e200165. doi: 10.1148/ryai.2021200165. eCollection 2021 May.	Astuto B	Radiol Artif Intell	2021	2021/06/18	PMC8166108		10.1148/ryai.2021200165
34324223	Deep learning-based segmentation of knee MRI for fully automatic subregional morphological assessment of cartilage tissues: Data from the Osteoarthritis Initiative	Panfilov E, Tiulpin A, Nieminen MT, Saarakkala S, Casula V.	J Orthop Res. 2022 May;40(5):1113-1124. doi: 10.1002/jor.25150. Epub 2021 Aug 6.	Panfilov E	J Orthop Res	2022	2021/07/29			10.1002/jor.25150
35726103	Deep learning to detect anterior cruciate ligament tear on knee MRI: multi-continental external validation	Tran A, Lassalle L, Zille P, Guillin R, Pluot E, Adam C, Charachon M, Brat H, Wallaert M, d'Assignies G, Rizk B.	Eur Radiol. 2022 Dec;32(12):8394-8403. doi: 10.1007/s00330-022-08923-z. Epub 2022 Jun 21.	Tran A	Eur Radiol	2022	2022/06/21			10.1007/s00330-022-08923-z
32755163	Using Deep Learning to Accelerate Knee MRI at 3 T: Results of an Interchangeability Study	Recht MP, Zbontar J, Sodickson DK, Knoll F, Yakubova N, Sriram A, Murrell T, Defazio A, Rabbat M, Rybak L, Kline M, Ciavarra G, Alaia EF, Samim M, Walter WR, Lin DJ, Lui YW, Muckley M, Huang Z, Johnson P, Stern R, Zitnick CL.	AJR Am J Roentgenol. 2020 Dec;215(6):1421-1429. doi: 10.2214/AJR.20.23313. Epub 2020 Oct 14.	Recht MP	AJR Am J Roentgenol	2020	2020/08/07	PMC8209682	NIHMS1707476	10.2214/AJR.20.23313
34663440	A deep learning method for predicting knee osteoarthritis radiographic progression from MRI	Schiratti JB, Dubois R, Herent P, Cahané D, Dachary J, Clozel T, Wainrib G, Keime-Guibert F, Lalande A, Pueyo M, Guillier R, Gabarroca C, Moingeon P.	Arthritis Res Ther. 2021 Oct 18;23(1):262. doi: 10.1186/s13075-021-02634-4.	Schiratti JB	Arthritis Res Ther	2021	2021/10/19	PMC8521982		10.1186/s13075-021-02634-4
31877380	Osteoarthritis year in review 2019: imaging	Kijowski R, Demehri S, Roemer F, Guermazi A.	Osteoarthritis Cartilage. 2020 Mar;28(3):285-295. doi: 10.1016/j.joca.2019.11.009. Epub 2019 Dec 23.	Kijowski R	Osteoarthritis Cartilage	2020	2019/12/27			10.1016/j.joca.2019.11.009
31755191	Rapid Knee MRI Acquisition and Analysis Techniques for Imaging Osteoarthritis	Chaudhari AS, Kogan F, Pedoia V, Majumdar S, Gold GE, Hargreaves BA.	J Magn Reson Imaging. 2020 Nov;52(5):1321-1339. doi: 10.1002/jmri.26991. Epub 2019 Nov 21.	Chaudhari AS	J Magn Reson Imaging	2020	2019/11/23	PMC7925938	NIHMS1670032	10.1002/jmri.26991
34567478	Deep Learning-Based Image Feature with Arthroscopy-Aided Early Diagnosis and Treatment of Meniscus Injury of Knee Joint	Li Z, Ren S, Zhang X, Bai L, Jiang C, Wu J, Zhang W.	J Healthc Eng. 2021 Sep 17;2021:2254594. doi: 10.1155/2021/2254594. eCollection 2021.	Li Z	J Healthc Eng	2021	2021/09/27	PMC8463205		10.1155/2021/2254594
30481176	Deep-learning-assisted diagnosis for knee magnetic resonance imaging: Development and retrospective validation of MRNet	Bien N, Rajpurkar P, Ball RL, Irvin J, Park A, Jones E, Bereket M, Patel BN, Yeom KW, Shpanskaya K, Halabi S, Zucker E, Fanton G, Amanatullah DF, Beaulieu CF, Riley GM, Stewart RJ, Blankenberg FG, Larson DB, Jones RH, Langlotz CP, Ng AY, Lungren MP.	PLoS Med. 2018 Nov 27;15(11):e1002699. doi: 10.1371/journal.pmed.1002699. eCollection 2018 Nov.	Bien N	PLoS Med	2018	2018/11/28	PMC6258509		10.1371/journal.pmed.1002699
33714850	Meniscal lesion detection and characterization in adult knee MRI: A deep learning model approach with external validation	Rizk B, Brat H, Zille P, Guillin R, Pouchy C, Adam C, Ardon R, d'Assignies G.	Phys Med. 2021 Mar;83:64-71. doi: 10.1016/j.ejmp.2021.02.010. Epub 2021 Mar 11.	Rizk B	Phys Med	2021	2021/03/14			10.1016/j.ejmp.2021.02.010
34211738	Applicability of deep learning-based reconstruction trained by brain and knee 3T MRI to lumbar 1.5T MRI	Kashiwagi N, Tanaka H, Yamashita Y, Takahashi H, Kassai Y, Fujiwara M, Tomiyama N.	Acta Radiol Open. 2021 Jun 18;10(6):20584601211023939. doi: 10.1177/20584601211023939. eCollection 2021 Jun.	Kashiwagi N	Acta Radiol Open	2021	2021/07/02	PMC8216362		10.1177/20584601211023939
37151912	Magnetic resonance imaging assessments for knee segmentation and their use in combination with machine/deep learning as predictors of early osteoarthritis diagnosis and prognosis	Martel-Pelletier J, Paiement P, Pelletier JP.	Ther Adv Musculoskelet Dis. 2023 Apr 28;15:1759720X231165560. doi: 10.1177/1759720X231165560. eCollection 2023.	Martel-Pelletier J	Ther Adv Musculoskelet Dis	2023	2023/05/08	PMC10155034		10.1177/1759720X231165560
35852498	Generalizability of Deep Learning Segmentation Algorithms for Automated Assessment of Cartilage Morphology and MRI Relaxometry	Schmidt AM, Desai AD, Watkins LE, Crowder HA, Black MS, Mazzoli V, Rubin EB, Lu Q, MacKay JW, Boutin RD, Kogan F, Gold GE, Hargreaves BA, Chaudhari AS.	J Magn Reson Imaging. 2023 Apr;57(4):1029-1039. doi: 10.1002/jmri.28365. Epub 2022 Jul 19.	Schmidt AM	J Magn Reson Imaging	2023	2022/07/19	PMC9849481	NIHMS1822687	10.1002/jmri.28365
34347157	Deep learning in knee imaging: a systematic review utilizing a Checklist for Artificial Intelligence in Medical Imaging (CLAIM)	Si L, Zhong J, Huo J, Xuan K, Zhuang Z, Hu Y, Wang Q, Zhang H, Yao W.	Eur Radiol. 2022 Feb;32(2):1353-1361. doi: 10.1007/s00330-021-08190-4. Epub 2021 Aug 4.	Si L	Eur Radiol	2022	2021/08/04			10.1007/s00330-021-08190-4
33331995	Automated age estimation of young individuals based on 3D knee MRI using deep learning	Mauer MA, Well EJ, Herrmann J, Groth M, Morlock MM, Maas R, Säring D.	Int J Legal Med. 2021 Mar;135(2):649-663. doi: 10.1007/s00414-020-02465-z. Epub 2020 Dec 17.	Mauer MA	Int J Legal Med	2021	2020/12/17	PMC7870623		10.1007/s00414-020-02465-z
34157062	Erratum: Automatic Deep Learning-assisted Detection and Grading of Abnormalities in Knee MRI Studies	Astuto B, Flament I, Namiri NK, Shah R, Bharadwaj U, Link TM, Bucknor MD, Pedoia V, Majumdar S.	Radiol Artif Intell. 2021 May 19;3(3):e219001. doi: 10.1148/ryai.2021219001. eCollection 2021 May.	Astuto B	Radiol Artif Intell	2021	2021/06/22	PMC8166114		10.1148/ryai.2021219001
35725760	Diagnostic advantage of thin slice 2D MRI and multiplanar reconstruction of the knee joint using deep learning based denoising approach	Kakigi T, Sakamoto R, Tagawa H, Kuriyama S, Goto Y, Nambu M, Sagawa H, Numamoto H, Miyake KK, Saga T, Matsuda S, Nakamoto Y.	Sci Rep. 2022 Jun 20;12(1):10362. doi: 10.1038/s41598-022-14190-1.	Kakigi T	Sci Rep	2022	2022/06/20	PMC9209466		10.1038/s41598-022-14190-1
36328943	End-to-end deep learning model for segmentation and severity staging of anterior cruciate ligament injuries from MRI	Dung NT, Thuan NH, Van Dung T, Van Nho L, Tri NM, Vy VPT, Hoang LN, Phat NT, Chuong DA, Dang LH.	Diagn Interv Imaging. 2023 Mar;104(3):133-141. doi: 10.1016/j.diii.2022.10.010. Epub 2022 Oct 31.	Dung NT	Diagn Interv Imaging	2023	2022/11/03			10.1016/j.diii.2022.10.010
35389046	Feasibility of an accelerated 2D-multi-contrast knee MRI protocol using deep-learning image reconstruction: a prospective intraindividual comparison with a standard MRI protocol	Herrmann J, Keller G, Gassenmaier S, Nickel D, Koerzdoerfer G, Mostapha M, Almansour H, Afat S, Othman AE.	Eur Radiol. 2022 Sep;32(9):6215-6229. doi: 10.1007/s00330-022-08753-z. Epub 2022 Apr 7.	Herrmann J	Eur Radiol	2022	2022/04/07	PMC9381615		10.1007/s00330-022-08753-z
35373388	High fidelity deep learning-based MRI reconstruction with instance-wise discriminative feature matching loss	Wang K, Tamir JI, De Goyeneche A, Wollner U, Brada R, Yu SX, Lustig M.	Magn Reson Med. 2022 Jul;88(1):476-491. doi: 10.1002/mrm.29227. Epub 2022 Apr 3.	Wang K	Magn Reson Med	2022	2022/04/04			10.1002/mrm.29227
35698440	Fully Automatic Knee Joint Segmentation and Quantitative Analysis for Osteoarthritis from Magnetic Resonance (MR) Images Using a Deep Learning Model	Tang X, Guo D, Liu A, Wu D, Liu J, Xu N, Qin Y.	Med Sci Monit. 2022 Jun 14;28:e936733. doi: 10.12659/MSM.936733.	Tang X	Med Sci Monit	2022	2022/06/14	PMC9206408		10.12659/MSM.936733
36791514	Auto-segmentation of the tibia and femur from knee MR images via deep learning and its application to cartilage strain and recovery	Kim-Wang SY, Bradley PX, Cutcliffe HC, Collins AT, Crook BS, Paranjape CS, Spritzer CE, DeFrate LE.	J Biomech. 2023 Mar;149:111473. doi: 10.1016/j.jbiomech.2023.111473. Epub 2023 Jan 26.	Kim-Wang SY	J Biomech	2023	2023/02/15	PMC10281551	NIHMS1873960	10.1016/j.jbiomech.2023.111473
33897991	DL-MRI: A Unified Framework of Deep Learning-Based MRI Super Resolution	Liu H, Liu J, Li J, Pan JS, Yu X.	J Healthc Eng. 2021 Apr 9;2021:5594649. doi: 10.1155/2021/5594649. eCollection 2021.	Liu H	J Healthc Eng	2021	2021/04/26	PMC8052167		10.1155/2021/5594649
35727152	Role of Thigh Muscle Changes in Knee Osteoarthritis Outcomes: Osteoarthritis Initiative Data	Mohajer B, Dolatshahi M, Moradi K, Najafzadeh N, Eng J, Zikria B, Wan M, Cao X, Roemer FW, Guermazi A, Demehri S.	Radiology. 2022 Oct;305(1):169-178. doi: 10.1148/radiol.212771. Epub 2022 Jun 21.	Mohajer B	Radiology	2022	2022/06/21	PMC9524577		10.1148/radiol.212771
34496667	Open Source Software for Automatic Subregional Assessment of Knee Cartilage Degradation Using Quantitative T2 Relaxometry and Deep Learning	Thomas KA, Krzemiński D, Kidziński Ł, Paul R, Rubin EB, Halilaj E, Black MS, Chaudhari A, Gold GE, Delp SL.	Cartilage. 2021 Dec;13(1_suppl):747S-756S. doi: 10.1177/19476035211042406. Epub 2021 Sep 8.	Thomas KA	Cartilage	2021	2021/09/09	PMC8808775		10.1177/19476035211042406
33973737	Subchondral Bone Length in Knee Osteoarthritis: A Deep Learning-Derived Imaging Measure and Its Association With Radiographic and Clinical Outcomes	Chang GH, Park LK, Le NA, Jhun RS, Surendran T, Lai J, Seo H, Promchotichai N, Yoon G, Scalera J, Capellini TD, Felson DT, Kolachalama VB.	Arthritis Rheumatol. 2021 Dec;73(12):2240-2248. doi: 10.1002/art.41808. Epub 2021 Oct 29.	Chang GH	Arthritis Rheumatol	2021	2021/05/11	PMC8581065	NIHMS1703971	10.1002/art.41808
36044484	One-Dimensional Deep Low-Rank and Sparse Network for Accelerated MRI	Wang Z, Qian C, Guo D, Sun H, Li R, Zhao B, Qu X.	IEEE Trans Med Imaging. 2023 Jan;42(1):79-90. doi: 10.1109/TMI.2022.3203312. Epub 2022 Dec 29.	Wang Z	IEEE Trans Med Imaging	2023	2022/08/31			10.1109/TMI.2022.3203312
35870296	Deep learning solution for medical image localization and orientation detection	Zhao Y, Zeng K, Zhao Y, Bhatia P, Ranganath M, Kozhikkavil ML, Li C, Hermosillo G.	Med Image Anal. 2022 Oct;81:102529. doi: 10.1016/j.media.2022.102529. Epub 2022 Jul 6.	Zhao Y	Med Image Anal	2022	2022/07/23			10.1016/j.media.2022.102529
35795003	Alternating Learning Approach for Variational Networks and Undersampling Pattern in Parallel MRI Applications	Zibetti MVW, Knoll F, Regatte RR.	IEEE Trans Comput Imaging. 2022;8:449-461. doi: 10.1109/tci.2022.3176129. Epub 2022 May 20.	Zibetti MVW	IEEE Trans Comput Imaging	2022	2022/07/07	PMC9252023	NIHMS1815214	10.1109/tci.2022.3176129
29582464	Super-resolution musculoskeletal MRI using deep learning	Chaudhari AS, Fang Z, Kogan F, Wood J, Stevens KJ, Gibbons EK, Lee JH, Gold GE, Hargreaves BA.	Magn Reson Med. 2018 Nov;80(5):2139-2154. doi: 10.1002/mrm.27178. Epub 2018 Mar 26.	Chaudhari AS	Magn Reson Med	2018	2018/03/28	PMC6107420	NIHMS949716	10.1002/mrm.27178
36564952	Editorial for "Deep-Learning-Based Contrast Synthesis From MRF Parameter Maps in the Knee Joint"	Singh A.	J Magn Reson Imaging. 2023 Aug;58(2):569-570. doi: 10.1002/jmri.28575. Epub 2022 Dec 23.	Singh A	J Magn Reson Imaging	2023	2022/12/24			10.1002/jmri.28575
36562500	Deep-Learning-Based Contrast Synthesis From MRF Parameter Maps in the Knee Joint	Nykänen O, Nevalainen M, Casula V, Isosalo A, Inkinen SI, Nikki M, Lattanzi R, Cloos MA, Nissi MJ, Nieminen MT.	J Magn Reson Imaging. 2023 Aug;58(2):559-568. doi: 10.1002/jmri.28573. Epub 2022 Dec 23.	Nykänen O	J Magn Reson Imaging	2023	2022/12/23	PMC10287835	NIHMS1856970	10.1002/jmri.28573
34035386	Deep learning for large scale MRI-based morphological phenotyping of osteoarthritis	Namiri NK, Lee J, Astuto B, Liu F, Shah R, Majumdar S, Pedoia V.	Sci Rep. 2021 May 25;11(1):10915. doi: 10.1038/s41598-021-90292-6.	Namiri NK	Sci Rep	2021	2021/05/26	PMC8149826		10.1038/s41598-021-90292-6
34138986	Putting the Pieces Together: Deep Learning for Knee MRI Multitissue Abnormality Detection and Severity Grading	D Li M, Y Chang C.	Radiol Artif Intell. 2021 Apr 14;3(3):e210022. doi: 10.1148/ryai.2021210022. eCollection 2021 May.	D Li M	Radiol Artif Intell	2021	2021/06/17	PMC8204128		10.1148/ryai.2021210022
35792956	Ensemble deep learning model for predicting anterior cruciate ligament tear from lateral knee radiograph	Kim DH, Chai JW, Kang JH, Lee JH, Kim HJ, Seo J, Choi JW.	Skeletal Radiol. 2022 Dec;51(12):2269-2279. doi: 10.1007/s00256-022-04081-x. Epub 2022 Jun 9.	Kim DH	Skeletal Radiol	2022	2022/07/06			10.1007/s00256-022-04081-x
31804183	Age Assessment of Youth and Young Adults Using Magnetic Resonance Imaging of the Knee: A Deep Learning Approach	Dallora AL, Berglund JS, Brogren M, Kvist O, Diaz Ruiz S, Dübbel A, Anderberg P.	JMIR Med Inform. 2019 Dec 5;7(4):e16291. doi: 10.2196/16291.	Dallora AL	JMIR Med Inform	2019	2019/12/06	PMC6923761		10.2196/16291
36624404	Acceleration of knee magnetic resonance imaging using a combination of compressed sensing and commercially available deep learning reconstruction: a preliminary study	Akai H, Yasaka K, Sugawara H, Tajima T, Kamitani M, Furuta T, Akahane M, Yoshioka N, Ohtomo K, Abe O, Kiryu S.	BMC Med Imaging. 2023 Jan 9;23(1):5. doi: 10.1186/s12880-023-00962-2.	Akai H	BMC Med Imaging	2023	2023/01/09	PMC9827641		10.1186/s12880-023-00962-2
35752117	Deep collaborative network with alpha matte for precise knee tissue segmentation from MRI	Khan S, Azam B, Yao Y, Chen W.	Comput Methods Programs Biomed. 2022 Jul;222:106963. doi: 10.1016/j.cmpb.2022.106963. Epub 2022 Jun 17.	Khan S	Comput Methods Programs Biomed	2022	2022/06/25			10.1016/j.cmpb.2022.106963
34003757	Interpretable and Lightweight 3-D Deep Learning Model for Automated ACL Diagnosis	Jeon Y, Yoshino K, Hagiwara S, Watanabe A, Quek ST, Yoshioka H, Feng M.	IEEE J Biomed Health Inform. 2021 Jul;25(7):2388-2397. doi: 10.1109/JBHI.2021.3081355. Epub 2021 Jul 27.	Jeon Y	IEEE J Biomed Health Inform	2021	2021/05/18			10.1109/JBHI.2021.3081355
34441418	Feasibility and Implementation of a Deep Learning MR Reconstruction for TSE Sequences in Musculoskeletal Imaging	Herrmann J, Koerzdoerfer G, Nickel D, Mostapha M, Nadar M, Gassenmaier S, Kuestner T, Othman AE.	Diagnostics (Basel). 2021 Aug 16;11(8):1484. doi: 10.3390/diagnostics11081484.	Herrmann J	Diagnostics (Basel)	2021	2021/08/27	PMC8394583		10.3390/diagnostics11081484
36372871	Automated meniscus segmentation and tear detection of knee MRI with a 3D mask-RCNN	Li YZ, Wang Y, Fang KB, Zheng HZ, Lai QQ, Xia YF, Chen JY, Dai ZS.	Eur J Med Res. 2022 Nov 14;27(1):247. doi: 10.1186/s40001-022-00883-w.	Li YZ	Eur J Med Res	2022	2022/11/14	PMC9661774		10.1186/s40001-022-00883-w
35669917	Knee Bone and Cartilage Segmentation Based on a 3D Deep Neural Network Using Adversarial Loss for Prior Shape Constraint	Chen H, Zhao N, Tan T, Kang Y, Sun C, Xie G, Verdonschot N, Sprengers A.	Front Med (Lausanne). 2022 May 20;9:792900. doi: 10.3389/fmed.2022.792900. eCollection 2022.	Chen H	Front Med (Lausanne)	2022	2022/06/07	PMC9163741		10.3389/fmed.2022.792900
37122858	Transfer learning-assisted 3D deep learning models for knee osteoarthritis detection: Data from the osteoarthritis initiative	Yeoh PSQ, Lai KW, Goh SL, Hasikin K, Wu X, Li P.	Front Bioeng Biotechnol. 2023 Apr 13;11:1164655. doi: 10.3389/fbioe.2023.1164655. eCollection 2023.	Yeoh PSQ	Front Bioeng Biotechnol	2023	2023/05/01	PMC10136763		10.3389/fbioe.2023.1164655
32793889	Deep Learning for Hierarchical Severity Staging of Anterior Cruciate Ligament Injuries from MRI	Namiri NK, Flament I, Astuto B, Shah R, Tibrewala R, Caliva F, Link TM, Pedoia V, Majumdar S.	Radiol Artif Intell. 2020 Jul 29;2(4):e190207. doi: 10.1148/ryai.2020190207.	Namiri NK	Radiol Artif Intell	2020	2020/08/15	PMC7392061		10.1148/ryai.2020190207
35620201	Automated Detection Model Based on Deep Learning for Knee Joint Motion Injury due to Martial Arts	Xue M, Liu Y, Cai X.	Comput Math Methods Med. 2022 May 17;2022:3647152. doi: 10.1155/2022/3647152. eCollection 2022.	Xue M	Comput Math Methods Med	2022	2022/05/27	PMC9129942		10.1155/2022/3647152
32168039	Deep Convolutional Neural Network-Based Diagnosis of Anterior Cruciate Ligament Tears: Performance Comparison of Homogenous Versus Heterogeneous Knee MRI Cohorts With Different Pulse Sequence Protocols and 1.5-T and 3-T Magnetic Field Strengths	Germann C, Marbach G, Civardi F, Fucentese SF, Fritz J, Sutter R, Pfirrmann CWA, Fritz B.	Invest Radiol. 2020 Aug;55(8):499-506. doi: 10.1097/RLI.0000000000000664.	Germann C	Invest Radiol	2020	2020/03/14	PMC7343178		10.1097/RLI.0000000000000664
30063195	Deep Learning Approach for Evaluating Knee MR Images: Achieving High Diagnostic Performance for Cartilage Lesion Detection	Liu F, Zhou Z, Samsonov A, Blankenbaker D, Larison W, Kanarek A, Lian K, Kambhampati S, Kijowski R.	Radiology. 2018 Oct;289(1):160-169. doi: 10.1148/radiol.2018172986. Epub 2018 Jul 31.	Liu F	Radiology	2018	2018/08/01	PMC6166867	NIHMS1001567	10.1148/radiol.2018172986
31793071	Automated cartilage and meniscus segmentation of knee MRI with conditional generative adversarial networks	Gaj S, Yang M, Nakamura K, Li X.	Magn Reson Med. 2020 Jul;84(1):437-449. doi: 10.1002/mrm.28111. Epub 2019 Dec 2.	Gaj S	Magn Reson Med	2020	2019/12/04			10.1002/mrm.28111
32286452	Deep Learning Predicts Total Knee Replacement from Magnetic Resonance Images	Tolpadi AA, Lee JJ, Pedoia V, Majumdar S.	Sci Rep. 2020 Apr 14;10(1):6371. doi: 10.1038/s41598-020-63395-9.	Tolpadi AA	Sci Rep	2020	2020/04/15	PMC7156761		10.1038/s41598-020-63395-9
35178233	A Torn ACL Mapping in Knee MRI Images Using Deep Convolution Neural Network with Inception-v3	Sridhar S, Amutharaj J, Valsalan P, Arthi B, Ramkumar S, Mathupriya S, Rajendran T, Waji YA.	J Healthc Eng. 2022 Feb 8;2022:7872500. doi: 10.1155/2022/7872500. eCollection 2022.	Sridhar S	J Healthc Eng	2022	2022/02/18	PMC8846973		10.1155/2022/7872500
32593389	A review on segmentation of knee articular cartilage: from conventional methods towards deep learning	Ebrahimkhani S, Jaward MH, Cicuttini FM, Dharmaratne A, Wang Y, de Herrera AGS.	Artif Intell Med. 2020 Jun;106:101851. doi: 10.1016/j.artmed.2020.101851. Epub 2020 May 6.	Ebrahimkhani S	Artif Intell Med	2020	2020/06/29			10.1016/j.artmed.2020.101851
33350717	A Deep Learning System for Synthetic Knee Magnetic Resonance Imaging: Is Artificial Intelligence-Based Fat-Suppressed Imaging Feasible?	Fayad LM, Parekh VS, de Castro Luna R, Ko CC, Tank D, Fritz J, Ahlawat S, Jacobs MA.	Invest Radiol. 2021 Jun 1;56(6):357-368. doi: 10.1097/RLI.0000000000000751.	Fayad LM	Invest Radiol	2021	2020/12/22	PMC8087629	NIHMS1644518	10.1097/RLI.0000000000000751
36016997	Automated detection of knee cystic lesions on magnetic resonance imaging using deep learning	Xiongfeng T, Yingzhi L, Xianyue S, Meng H, Bo C, Deming G, Yanguo Q.	Front Med (Lausanne). 2022 Aug 9;9:928642. doi: 10.3389/fmed.2022.928642. eCollection 2022.	Xiongfeng T	Front Med (Lausanne)	2022	2022/08/26	PMC9397605		10.3389/fmed.2022.928642
33724507	Analysis of deep complex-valued convolutional neural networks for MRI reconstruction and phase-focused applications	Cole E, Cheng J, Pauly J, Vasanawala S.	Magn Reson Med. 2021 Aug;86(2):1093-1109. doi: 10.1002/mrm.28733. Epub 2021 Mar 16.	Cole E	Magn Reson Med	2021	2021/03/16	PMC8291740	NIHMS1669248	10.1002/mrm.28733
35502381	Automated knee cartilage segmentation for heterogeneous clinical MRI using generative adversarial networks with transfer learning	Yang M, Colak C, Chundru KK, Gaj S, Nanavati A, Jones MH, Winalski CS, Subhas N, Li X.	Quant Imaging Med Surg. 2022 May;12(5):2620-2633. doi: 10.21037/qims-21-459.	Yang M	Quant Imaging Med Surg	2022	2022/05/03	PMC9014147		10.21037/qims-21-459
35847603	Identification and diagnosis of meniscus tear by magnetic resonance imaging using a deep learning model	Li J, Qian K, Liu J, Huang Z, Zhang Y, Zhao G, Wang H, Li M, Liang X, Zhou F, Yu X, Li L, Wang X, Yang X, Jiang Q.	J Orthop Translat. 2022 Jun 26;34:91-101. doi: 10.1016/j.jot.2022.05.006. eCollection 2022 May.	Li J	J Orthop Translat	2022	2022/07/18	PMC9253363		10.1016/j.jot.2022.05.006
31872357	Clinical evaluation of fully automated thigh muscle and adipose tissue segmentation using a U-Net deep learning architecture in context of osteoarthritic knee pain	Kemnitz J, Baumgartner CF, Eckstein F, Chaudhari A, Ruhdorfer A, Wirth W, Eder SK, Konukoglu E.	MAGMA. 2020 Aug;33(4):483-493. doi: 10.1007/s10334-019-00816-5. Epub 2019 Dec 23.	Kemnitz J	MAGMA	2020	2019/12/25	PMC7351818		10.1007/s10334-019-00816-5
35811127	Commercially Available Deep-learning-reconstruction of MR Imaging of the Knee at 1.5T Has Higher Image Quality Than Conventionally-reconstructed Imaging at 3T: A Normal Volunteer Study	Akai H, Yasaka K, Sugawara H, Tajima T, Akahane M, Yoshioka N, Ohtomo K, Abe O, Kiryu S.	Magn Reson Med Sci. 2023 Jul 1;22(3):353-360. doi: 10.2463/mrms.mp.2022-0020. Epub 2022 Jul 9.	Akai H	Magn Reson Med Sci	2023	2022/07/10	PMC10449552		10.2463/mrms.mp.2022-0020
36527935	A Joint Group Sparsity-based deep learning for multi-contrast MRI reconstruction	Guo D, Zeng G, Fu H, Wang Z, Yang Y, Qu X.	J Magn Reson. 2023 Jan;346:107354. doi: 10.1016/j.jmr.2022.107354. Epub 2022 Dec 8.	Guo D	J Magn Reson	2023	2022/12/17			10.1016/j.jmr.2022.107354
37547408	MGACA-Net: a novel deep learning based multi-scale guided attention and context aggregation for localization of knee anterior cruciate ligament tears region in MRI images	Awan MJ, Mohd Rahim MS, Salim N, Nobanee H, Asif AA, Attiq MO.	PeerJ Comput Sci. 2023 Jul 13;9:e1483. doi: 10.7717/peerj-cs.1483. eCollection 2023.	Awan MJ	PeerJ Comput Sci	2023	2023/08/07	PMC10403161		10.7717/peerj-cs.1483
35759870	Automatic Grading Assessments for Knee MRI Cartilage Defects via Self-ensembling Semi-supervised Learning with Dual-Consistency	Huo J, Ouyang X, Si L, Xuan K, Wang S, Yao W, Liu Y, Xu J, Qian D, Xue Z, Wang Q, Shen D, Zhang L.	Med Image Anal. 2022 Aug;80:102508. doi: 10.1016/j.media.2022.102508. Epub 2022 Jun 18.	Huo J	Med Image Anal	2022	2022/06/27			10.1016/j.media.2022.102508
34926416	A Multi-Task Deep Learning Method for Detection of Meniscal Tears in MRI Data from the Osteoarthritis Initiative Database	Tack A, Shestakov A, Lüdke D, Zachow S.	Front Bioeng Biotechnol. 2021 Dec 2;9:747217. doi: 10.3389/fbioe.2021.747217. eCollection 2021.	Tack A	Front Bioeng Biotechnol	2021	2021/12/20	PMC8675251		10.3389/fbioe.2021.747217
35678739	Simultaneously optimizing sampling pattern for joint acceleration of multi-contrast MRI using model-based deep learning	Seo S, Luu HM, Choi SH, Park SH.	Med Phys. 2022 Sep;49(9):5964-5980. doi: 10.1002/mp.15790. Epub 2022 Jun 20.	Seo S	Med Phys	2022	2022/06/09			10.1002/mp.15790
24579147	Deep feature learning for knee cartilage segmentation using a triplanar convolutional neural network	Prasoon A, Petersen K, Igel C, Lauze F, Dam E, Nielsen M.	Med Image Comput Comput Assist Interv. 2013;16(Pt 2):246-53. doi: 10.1007/978-3-642-40763-5_31.	Prasoon A	Med Image Comput Comput Assist Interv	2013	2014/03/01			10.1007/978-3-642-40763-5_31
28670152	Deep Learning in Medical Imaging: General Overview	Lee JG, Jun S, Cho YW, Lee H, Kim GB, Seo JB, Kim N.	Korean J Radiol. 2017 Jul-Aug;18(4):570-584. doi: 10.3348/kjr.2017.18.4.570. Epub 2017 May 19.	Lee JG	Korean J Radiol	2017	2017/07/04	PMC5447633		10.3348/kjr.2017.18.4.570
31958580	Semi-supervised learning for automatic segmentation of the knee from MRI with convolutional neural networks	Burton W 2nd, Myers C, Rullkoetter P.	Comput Methods Programs Biomed. 2020 Jun;189:105328. doi: 10.1016/j.cmpb.2020.105328. Epub 2020 Jan 11.	Burton W 2nd	Comput Methods Programs Biomed	2020	2020/01/21			10.1016/j.cmpb.2020.105328
33440798	Efficient Detection of Knee Anterior Cruciate Ligament from Magnetic Resonance Imaging Using Deep Learning Approach	Awan MJ, Rahim MSM, Salim N, Mohammed MA, Garcia-Zapirain B, Abdulkareem KH.	Diagnostics (Basel). 2021 Jan 11;11(1):105. doi: 10.3390/diagnostics11010105.	Awan MJ	Diagnostics (Basel)	2021	2021/01/14	PMC7826961		10.3390/diagnostics11010105
31582263	Super-resolution reconstruction of knee magnetic resonance imaging based on deep learning	Qiu D, Zhang S, Liu Y, Zhu J, Zheng L.	Comput Methods Programs Biomed. 2020 Apr;187:105059. doi: 10.1016/j.cmpb.2019.105059. Epub 2019 Sep 24.	Qiu D	Comput Methods Programs Biomed	2020	2019/10/05			10.1016/j.cmpb.2019.105059
30273968	Translation of morphological and functional musculoskeletal imaging	Pedoia V, Majumdar S.	J Orthop Res. 2019 Jan;37(1):23-34. doi: 10.1002/jor.24151. Epub 2018 Oct 29.	Pedoia V	J Orthop Res	2019	2018/10/02			10.1002/jor.24151
34735607	AI MSK clinical applications: cartilage and osteoarthritis	Joseph GB, McCulloch CE, Sohn JH, Pedoia V, Majumdar S, Link TM.	Skeletal Radiol. 2022 Feb;51(2):331-343. doi: 10.1007/s00256-021-03909-2. Epub 2021 Nov 4.	Joseph GB	Skeletal Radiol	2022	2021/11/04			10.1007/s00256-021-03909-2
32992372	Improving Quantitative Magnetic Resonance Imaging Using Deep Learning	Liu F.	Semin Musculoskelet Radiol. 2020 Aug;24(4):451-459. doi: 10.1055/s-0040-1709482. Epub 2020 Sep 29.	Liu F	Semin Musculoskelet Radiol	2020	2020/09/29	PMC8164439	NIHMS1700390	10.1055/s-0040-1709482
31535731	Knee menisci segmentation and relaxometry of 3D ultrashort echo time cones MR imaging using attention U-Net with transfer learning	Byra M, Wu M, Zhang X, Jang H, Ma YJ, Chang EY, Shah S, Du J.	Magn Reson Med. 2020 Mar;83(3):1109-1122. doi: 10.1002/mrm.27969. Epub 2019 Sep 19.	Byra M	Magn Reson Med	2020	2019/09/20	PMC6879791	NIHMS1045869	10.1002/mrm.27969
31621571	Magnetic resonance imaging assessment of knee osteoarthritis: current and developing new concepts and techniques	Hayashi D, Roemer FW, Guermazi A.	Clin Exp Rheumatol. 2019 Sep-Oct;37 Suppl 120(5):88-95. Epub 2019 Oct 15.	Hayashi D	Clin Exp Rheumatol	2019	2019/10/18			
36564430	Region of interest-specific loss functions improve T(2) quantification with ultrafast T(2) mapping MRI sequences in knee, hip and lumbar spine	Tolpadi AA, Han M, Calivà F, Pedoia V, Majumdar S.	Sci Rep. 2022 Dec 23;12(1):22208. doi: 10.1038/s41598-022-26266-z.	Tolpadi AA	Sci Rep	2022	2022/12/23	PMC9789075		10.1038/s41598-022-26266-z
32076658	Fully Automated Diagnosis of Anterior Cruciate Ligament Tears on Knee MR Images by Using Deep Learning	Liu F, Guan B, Zhou Z, Samsonov A, Rosas H, Lian K, Sharma R, Kanarek A, Kim J, Guermazi A, Kijowski R.	Radiol Artif Intell. 2019 May 8;1(3):180091. doi: 10.1148/ryai.2019180091.	Liu F	Radiol Artif Intell	2019	2020/02/21	PMC6542618		10.1148/ryai.2019180091
35524293	Automatic segmentation model of intercondylar fossa based on deep learning: a novel and effective assessment method for the notch volume	Li M, Bai H, Zhang F, Zhou Y, Lin Q, Zhou Q, Feng Q, Zhang L.	BMC Musculoskelet Disord. 2022 May 6;23(1):426. doi: 10.1186/s12891-022-05378-7.	Li M	BMC Musculoskelet Disord	2022	2022/05/07	PMC9074347		10.1186/s12891-022-05378-7
33634142	Knee Cartilage Thickness Differs Alongside Ages: A 3-T Magnetic Resonance Research Upon 2,481 Subjects via Deep Learning	Si L, Xuan K, Zhong J, Huo J, Xing Y, Geng J, Hu Y, Zhang H, Wang Q, Yao W.	Front Med (Lausanne). 2021 Feb 9;7:600049. doi: 10.3389/fmed.2020.600049. eCollection 2020.	Si L	Front Med (Lausanne)	2021	2021/02/26	PMC7900571		10.3389/fmed.2020.600049
36793660	Prediction Models for Knee Osteoarthritis: Review of Current Models and Future Directions	Ramazanian T, Fu S, Sohn S, Taunton MJ, Kremers HM.	Arch Bone Jt Surg. 2023;11(1):1-11. doi: 10.22038/ABJS.2022.58485.2897.	Ramazanian T	Arch Bone Jt Surg	2023	2023/02/16	PMC9903309		10.22038/ABJS.2022.58485.2897
36243308	The KNee OsteoArthritis Prediction (KNOAP2020) challenge: An image analysis challenge to predict incident symptomatic radiographic knee osteoarthritis from MRI and X-ray images	Hirvasniemi J, Runhaar J, van der Heijden RA, Zokaeinikoo M, Yang M, Li X, Tan J, Rajamohan HR, Zhou Y, Deniz CM, Caliva F, Iriondo C, Lee JJ, Liu F, Martinez AM, Namiri N, Pedoia V, Panfilov E, Bayramoglu N, Nguyen HH, Nieminen MT, Saarakkala S, Tiulpin A, Lin E, Li A, Li V, Dam EB, Chaudhari AS, Kijowski R, Bierma-Zeinstra S, Oei EHG, Klein S.	Osteoarthritis Cartilage. 2023 Jan;31(1):115-125. doi: 10.1016/j.joca.2022.10.001. Epub 2022 Oct 12.	Hirvasniemi J	Osteoarthritis Cartilage	2023	2022/10/15	PMC10323696	NIHMS1905689	10.1016/j.joca.2022.10.001
34087612	A deep cascade of ensemble of dual domain networks with gradient-based T1 assistance and perceptual refinement for fast MRI reconstruction	Murugesan B, Ramanarayanan S, Vijayarangan S, Ram K, Jagannathan NR, Sivaprakasam M.	Comput Med Imaging Graph. 2021 Jul;91:101942. doi: 10.1016/j.compmedimag.2021.101942. Epub 2021 May 24.	Murugesan B	Comput Med Imaging Graph	2021	2021/06/04			10.1016/j.compmedimag.2021.101942
36204533	RadImageNet: An Open Radiologic Deep Learning Research Dataset for Effective Transfer Learning	Mei X, Liu Z, Robson PM, Marinelli B, Huang M, Doshi A, Jacobi A, Cao C, Link KE, Yang T, Wang Y, Greenspan H, Deyer T, Fayad ZA, Yang Y.	Radiol Artif Intell. 2022 Jul 27;4(5):e210315. doi: 10.1148/ryai.210315. eCollection 2022 Sep.	Mei X	Radiol Artif Intell	2022	2022/10/07	PMC9530758		10.1148/ryai.210315
34938819	Three-Dimensional Magnetic Resonance Imaging Bone Models of the Hip Joint Using Deep Learning: Dynamic Simulation of Hip Impingement for Diagnosis of Intra- and Extra-articular Hip Impingement	Zeng G, Degonda C, Boschung A, Schmaranzer F, Gerber N, Siebenrock KA, Steppacher SD, Tannast M, Lerch TD.	Orthop J Sports Med. 2021 Nov 24;9(12):23259671211046916. doi: 10.1177/23259671211046916. eCollection 2021 Dec.	Zeng G	Orthop J Sports Med	2021	2021/12/23	PMC8685729		10.1177/23259671211046916
32162395	Fully automated and robust analysis technique for popliteal artery vessel wall evaluation (FRAPPE) using neural network models from standardized knee MRI	Chen L, Canton G, Liu W, Hippe DS, Balu N, Watase H, Hatsukami TS, Waterton JC, Hwang JN, Yuan C.	Magn Reson Med. 2020 Oct;84(4):2147-2160. doi: 10.1002/mrm.28237. Epub 2020 Mar 11.	Chen L	Magn Reson Med	2020	2020/03/13	PMC8320767	NIHMS1570342	10.1002/mrm.28237
32956045	Uncertainty Quantification in Deep MRI Reconstruction	Edupuganti V, Mardani M, Vasanawala S, Pauly J.	IEEE Trans Med Imaging. 2021 Jan;40(1):239-250. doi: 10.1109/TMI.2020.3025065. Epub 2020 Dec 29.	Edupuganti V	IEEE Trans Med Imaging	2021	2020/09/21	PMC7837266	NIHMS1658436	10.1109/TMI.2020.3025065
34918423	Cross-Cohort Automatic Knee MRI Segmentation With Multi-Planar U-Nets	Perslev M, Pai A, Runhaar J, Igel C, Dam EB.	J Magn Reson Imaging. 2022 Jun;55(6):1650-1663. doi: 10.1002/jmri.27978. Epub 2021 Dec 17.	Perslev M	J Magn Reson Imaging	2022	2021/12/17	PMC9106804	NIHMS1750740	10.1002/jmri.27978
36126403	KCB-Net: A 3D knee cartilage and bone segmentation network via sparse annotation	Peng Y, Zheng H, Liang P, Zhang L, Zaman F, Wu X, Sonka M, Chen DZ.	Med Image Anal. 2022 Nov;82:102574. doi: 10.1016/j.media.2022.102574. Epub 2022 Sep 7.	Peng Y	Med Image Anal	2022	2022/09/20			10.1016/j.media.2022.102574
30905742	Diagnosing osteoarthritis from T(2) maps using deep learning: an analysis of the entire Osteoarthritis Initiative baseline cohort	Pedoia V, Lee J, Norman B, Link TM, Majumdar S.	Osteoarthritis Cartilage. 2019 Jul;27(7):1002-1010. doi: 10.1016/j.joca.2019.02.800. Epub 2019 Mar 21.	Pedoia V	Osteoarthritis Cartilage	2019	2019/03/26	PMC6579664	NIHMS1525123	10.1016/j.joca.2019.02.800
35059693	Deep J-Sense: Accelerated MRI Reconstruction via Unrolled Alternating Optimization	Arvinte M, Vishwanath S, Tewfik AH, Tamir JI.	Med Image Comput Comput Assist Interv. 2021 Sep-Oct;12906:350-360. doi: 10.1007/978-3-030-87231-1_34. Epub 2021 Sep 21.	Arvinte M	Med Image Comput Comput Assist Interv	2021	2022/01/21	PMC8767765	NIHMS1765492	10.1007/978-3-030-87231-1_34
34062366	Transfer learning enhanced generative adversarial networks for multi-channel MRI reconstruction	Lv J, Li G, Tong X, Chen W, Huang J, Wang C, Yang G.	Comput Biol Med. 2021 Jul;134:104504. doi: 10.1016/j.compbiomed.2021.104504. Epub 2021 May 26.	Lv J	Comput Biol Med	2021	2021/06/01			10.1016/j.compbiomed.2021.104504
32759045	pFISTA-SENSE-ResNet for parallel MRI reconstruction	Lu T, Zhang X, Huang Y, Guo D, Huang F, Xu Q, Hu Y, Ou-Yang L, Lin J, Yan Z, Qu X.	J Magn Reson. 2020 Sep;318:106790. doi: 10.1016/j.jmr.2020.106790. Epub 2020 Jul 21.	Lu T	J Magn Reson	2020	2020/08/08			10.1016/j.jmr.2020.106790
30859341	Deep Learning for Detection of Complete Anterior Cruciate Ligament Tear	Chang PD, Wong TT, Rasiej MJ.	J Digit Imaging. 2019 Dec;32(6):980-986. doi: 10.1007/s10278-019-00193-4.	Chang PD	J Digit Imaging	2019	2019/03/13	PMC6841825		10.1007/s10278-019-00193-4
34306588	Deep Learning-Based Magnetic Resonance Imaging Image Features for Diagnosis of Anterior Cruciate Ligament Injury	Li Z, Ren S, Zhou R, Jiang X, You T, Li C, Zhang W.	J Healthc Eng. 2021 Jul 2;2021:4076175. doi: 10.1155/2021/4076175. eCollection 2021.	Li Z	J Healthc Eng	2021	2021/07/26	PMC8272672		10.1155/2021/4076175
36092783	Detection Method of Athlete Joint Injury Based on Deep Learning Model	Liu J, Yang X, Liao T, Huang Y.	Comput Math Methods Med. 2022 Sep 2;2022:8165580. doi: 10.1155/2022/8165580. eCollection 2022.	Liu J	Comput Math Methods Med	2022	2022/09/12	PMC9462975		10.1155/2022/8165580
34151253	Evaluation of MRI Denoising Methods Using Unsupervised Learning	Moreno López M, Frederick JM, Ventura J.	Front Artif Intell. 2021 Jun 4;4:642731. doi: 10.3389/frai.2021.642731. eCollection 2021.	Moreno López M	Front Artif Intell	2021	2021/06/21	PMC8212039		10.3389/frai.2021.642731
34892119	MRI Knee Domain Translation for Unsupervised Segmentation By CycleGAN (data from Osteoarthritis initiative (OAI))	Felfeliyan B, Hareendranathan A, Kuntze G, Jaremko J, Ronsky J.	Annu Int Conf IEEE Eng Med Biol Soc. 2021 Nov;2021:4052-4055. doi: 10.1109/EMBC46164.2021.9629705.	Felfeliyan B	Annu Int Conf IEEE Eng Med Biol Soc	2021	2021/12/11			10.1109/EMBC46164.2021.9629705
35192462	Pyramid Convolutional RNN for MRI Image Reconstruction	Chen EZ, Wang P, Chen X, Chen T, Sun S.	IEEE Trans Med Imaging. 2022 Aug;41(8):2033-2047. doi: 10.1109/TMI.2022.3153849. Epub 2022 Aug 1.	Chen EZ	IEEE Trans Med Imaging	2022	2022/02/22			10.1109/TMI.2022.3153849
35383186	fastMRI+, Clinical pathology annotations for knee and brain fully sampled magnetic resonance imaging data	Zhao R, Yaman B, Zhang Y, Stewart R, Dixon A, Knoll F, Huang Z, Lui YW, Hansen MS, Lungren MP.	Sci Data. 2022 Apr 5;9(1):152. doi: 10.1038/s41597-022-01255-z.	Zhao R	Sci Data	2022	2022/04/06	PMC8983757		10.1038/s41597-022-01255-z
29115689	Learning a variational network for reconstruction of accelerated MRI data	Hammernik K, Klatzer T, Kobler E, Recht MP, Sodickson DK, Pock T, Knoll F.	Magn Reson Med. 2018 Jun;79(6):3055-3071. doi: 10.1002/mrm.26977. Epub 2017 Nov 8.	Hammernik K	Magn Reson Med	2018	2017/11/09	PMC5902683	NIHMS928633	10.1002/mrm.26977
36716684	DSMENet: Detail and Structure Mutually Enhancing Network for under-sampled MRI reconstruction	Wang Y, Pang Y, Tong C.	Comput Biol Med. 2023 Mar;154:106204. doi: 10.1016/j.compbiomed.2022.106204. Epub 2022 Oct 13.	Wang Y	Comput Biol Med	2023	2023/01/30			10.1016/j.compbiomed.2022.106204
35713187	Deep learning-based quantitative susceptibility mapping (QSM) in the presence of fat using synthetically generated multi-echo phase training data	Hanspach J, Bollmann S, Grigo J, Karius A, Uder M, Laun FB.	Magn Reson Med. 2022 Oct;88(4):1548-1560. doi: 10.1002/mrm.29265. Epub 2022 Jun 17.	Hanspach J	Magn Reson Med	2022	2022/06/17			10.1002/mrm.29265
33231896	Robust water-fat separation based on deep learning model exploring multi-echo nature of mGRE	Liu K, Li X, Li Z, Chen Y, Xiong H, Chen F, Bao Q, Liu C.	Magn Reson Med. 2021 May;85(5):2828-2841. doi: 10.1002/mrm.28586. Epub 2020 Nov 24.	Liu K	Magn Reson Med	2021	2020/11/24			10.1002/mrm.28586
36651242	[Deep parallel MRI reconstruction based on a complex-valued loss function]	Huang J, Zhou G, Yu Z, Hu W.	Nan Fang Yi Ke Da Xue Xue Bao. 2022 Dec 20;42(12):1755-1764. doi: 10.12122/j.issn.1673-4254.2022.12.02.	Huang J	Nan Fang Yi Ke Da Xue Xue Bao	2022	2023/01/18	PMC9878414		10.12122/j.issn.1673-4254.2022.12.02
31732262	MR Protocol Optimization With Deep Learning: A Proof of Concept	Richardson ML.	Curr Probl Diagn Radiol. 2021 Mar-Apr;50(2):168-174. doi: 10.1067/j.cpradiol.2019.10.004. Epub 2019 Oct 21.	Richardson ML	Curr Probl Diagn Radiol	2021	2019/11/17			10.1067/j.cpradiol.2019.10.004
30529224	Automated segmentation of knee bone and cartilage combining statistical shape knowledge and convolutional neural networks: Data from the Osteoarthritis Initiative	Ambellan F, Tack A, Ehlke M, Zachow S.	Med Image Anal. 2019 Feb;52:109-118. doi: 10.1016/j.media.2018.11.009. Epub 2018 Nov 17.	Ambellan F	Med Image Anal	2019	2018/12/12			10.1016/j.media.2018.11.009
32879854	Diagnostic interchangeability of deep convolutional neural networks reconstructed knee MR images: preliminary experience	Subhas N, Li H, Yang M, Winalski CS, Polster J, Obuchowski N, Mamoto K, Liu R, Zhang C, Huang P, Gaire SK, Liang D, Shen B, Li X, Ying L.	Quant Imaging Med Surg. 2020 Sep;10(9):1748-1762. doi: 10.21037/qims-20-664.	Subhas N	Quant Imaging Med Surg	2020	2020/09/04	PMC7417759		10.21037/qims-20-664
36474467	Fast quantitative bone marrow lesion measurement on knee MRI for the assessment of osteoarthritis	Preiswerk F, Sury MS, Wortman JR, Neumann G, Wells W, Duryea J.	Osteoarthr Cartil Open. 2022 Jan 10;4(1):100234. doi: 10.1016/j.ocarto.2022.100234. eCollection 2022 Mar.	Preiswerk F	Osteoarthr Cartil Open	2022	2022/12/07	PMC9718203		10.1016/j.ocarto.2022.100234
35652115	MIMIR: Deep Regression for Automated Analysis of UK Biobank MRI Scans	Langner T, Martínez Mora A, Strand R, Ahlström H, Kullberg J.	Radiol Artif Intell. 2022 Apr 6;4(3):e210178. doi: 10.1148/ryai.210178. eCollection 2022 May.	Langner T	Radiol Artif Intell	2022	2022/06/02	PMC9152682		10.1148/ryai.210178
31373061	MRI Gibbs-ringing artifact reduction by means of machine learning using convolutional neural networks	Zhang Q, Ruan G, Yang W, Liu Y, Zhao K, Feng Q, Chen W, Wu EX, Feng Y.	Magn Reson Med. 2019 Dec;82(6):2133-2145. doi: 10.1002/mrm.27894. Epub 2019 Aug 2.	Zhang Q	Magn Reson Med	2019	2019/08/03			10.1002/mrm.27894
29774597	Assessment of the generalization of learned image reconstruction and the potential for transfer learning	Knoll F, Hammernik K, Kobler E, Pock T, Recht MP, Sodickson DK.	Magn Reson Med. 2019 Jan;81(1):116-128. doi: 10.1002/mrm.27355. Epub 2018 May 17.	Knoll F	Magn Reson Med	2019	2018/05/19	PMC6240410	NIHMS962219	10.1002/mrm.27355
28733975	Deep convolutional neural network and 3D deformable approach for tissue segmentation in musculoskeletal magnetic resonance imaging	Liu F, Zhou Z, Jang H, Samsonov A, Zhao G, Kijowski R.	Magn Reson Med. 2018 Apr;79(4):2379-2391. doi: 10.1002/mrm.26841. Epub 2017 Jul 21.	Liu F	Magn Reson Med	2018	2017/07/23	PMC6271435	NIHMS996920	10.1002/mrm.26841
32055951	Assessment of knee pain from MR imaging using a convolutional Siamese network	Chang GH, Felson DT, Qiu S, Guermazi A, Capellini TD, Kolachalama VB.	Eur Radiol. 2020 Jun;30(6):3538-3548. doi: 10.1007/s00330-020-06658-3. Epub 2020 Feb 13.	Chang GH	Eur Radiol	2020	2020/02/15	PMC7786238	NIHMS1561607	10.1007/s00330-020-06658-3
35214451	Automated Knee MR Images Segmentation of Anterior Cruciate Ligament Tears	Awan MJ, Rahim MSM, Salim N, Rehman A, Garcia-Zapirain B.	Sensors (Basel). 2022 Feb 17;22(4):1552. doi: 10.3390/s22041552.	Awan MJ	Sensors (Basel)	2022	2022/02/26	PMC8876207		10.3390/s22041552
36446308	Deep convolutional feature details for better knee disorder diagnoses in magnetic resonance images	Dunnhofer M, Martinel N, Micheloni C.	Comput Med Imaging Graph. 2022 Dec;102:102142. doi: 10.1016/j.compmedimag.2022.102142. Epub 2022 Nov 21.	Dunnhofer M	Comput Med Imaging Graph	2022	2022/11/29			10.1016/j.compmedimag.2022.102142
36049450	CAN3D: Fast 3D medical image segmentation via compact context aggregation	Dai W, Woo B, Liu S, Marques M, Engstrom C, Greer PB, Crozier S, Dowling JA, Chandra SS.	Med Image Anal. 2022 Nov;82:102562. doi: 10.1016/j.media.2022.102562. Epub 2022 Aug 9.	Dai W	Med Image Anal	2022	2022/09/01			10.1016/j.media.2022.102562
32980503	High-performance rapid MR parameter mapping using model-based deep adversarial learning	Liu F, Kijowski R, Feng L, El Fakhri G.	Magn Reson Imaging. 2020 Dec;74:152-160. doi: 10.1016/j.mri.2020.09.021. Epub 2020 Sep 25.	Liu F	Magn Reson Imaging	2020	2020/09/27	PMC7669737	NIHMS1634937	10.1016/j.mri.2020.09.021
34611904	Partial Fourier reconstruction of complex MR images using complex-valued convolutional neural networks	Xiao L, Liu Y, Yi Z, Zhao Y, Xie L, Cao P, Leong ATL, Wu EX.	Magn Reson Med. 2022 Feb;87(2):999-1014. doi: 10.1002/mrm.29033. Epub 2021 Oct 5.	Xiao L	Magn Reson Med	2022	2021/10/06			10.1002/mrm.29033
30188817	Learning Cross-Modality Representations From Multi-Modal Images	van Tulder G, de Bruijne M.	IEEE Trans Med Imaging. 2019 Feb;38(2):638-648. doi: 10.1109/TMI.2018.2868977. Epub 2018 Sep 6.	van Tulder G	IEEE Trans Med Imaging	2019	2018/09/07			10.1109/TMI.2018.2868977
35789133	Multi-mask self-supervised learning for physics-guided neural networks in highly accelerated magnetic resonance imaging	Yaman B, Gu H, Hosseini SAH, Demirel OB, Moeller S, Ellermann J, Uğurbil K, Akçakaya M.	NMR Biomed. 2022 Dec;35(12):e4798. doi: 10.1002/nbm.4798. Epub 2022 Jul 17.	Yaman B	NMR Biomed	2022	2022/07/05	PMC9669191	NIHMS1821616	10.1002/nbm.4798
32038862	Deep segmentation leverages geometric pose estimation in computer-aided total knee arthroplasty	Rodrigues P, Antunes M, Raposo C, Marques P, Fonseca F, Barreto JP.	Healthc Technol Lett. 2019 Dec 6;6(6):226-230. doi: 10.1049/htl.2019.0078. eCollection 2019 Dec.	Rodrigues P	Healthc Technol Lett	2019	2020/02/11	PMC6952257		10.1049/htl.2019.0078
35426470	Improving high frequency image features of deep learning reconstructions via k-space refinement with null-space kernel	Ryu K, Alkan C, Vasanawala SS.	Magn Reson Med. 2022 Sep;88(3):1263-1272. doi: 10.1002/mrm.29261. Epub 2022 Apr 15.	Ryu K	Magn Reson Med	2022	2022/04/15	PMC9246879	NIHMS1791972	10.1002/mrm.29261
35172227	Surface spherical encoding and contrastive learning for virtual bone shape aging	Calivá F, Kamat S, Morales Martinez A, Majumdar S, Pedoia V.	Med Image Anal. 2022 Apr;77:102388. doi: 10.1016/j.media.2022.102388. Epub 2022 Feb 7.	Calivá F	Med Image Anal	2022	2022/02/16			10.1016/j.media.2022.102388
30703579	Recurrent inference machines for reconstructing heterogeneous MRI data	Lønning K, Putzky P, Sonke JJ, Reneman L, Caan MWA, Welling M.	Med Image Anal. 2019 Apr;53:64-78. doi: 10.1016/j.media.2019.01.005. Epub 2019 Jan 18.	Lønning K	Med Image Anal	2019	2019/02/01			10.1016/j.media.2019.01.005
34441318	TransMed: Transformers Advance Multi-Modal Medical Image Classification	Dai Y, Gao Y, Liu F.	Diagnostics (Basel). 2021 Jul 31;11(8):1384. doi: 10.3390/diagnostics11081384.	Dai Y	Diagnostics (Basel)	2021	2021/08/27	PMC8391808		10.3390/diagnostics11081384
32243657	Learning osteoarthritis imaging biomarkers from bone surface spherical encoding	Morales Martinez A, Caliva F, Flament I, Liu F, Lee J, Cao P, Shah R, Majumdar S, Pedoia V.	Magn Reson Med. 2020 Oct;84(4):2190-2203. doi: 10.1002/mrm.28251. Epub 2020 Apr 3.	Morales Martinez A	Magn Reson Med	2020	2020/04/04	PMC7329596	NIHMS1582357	10.1002/mrm.28251
37426615	Medical imaging in rheumatoid arthritis: A review on deep learning approach	Parashar A, Rishi R, Parashar A, Rida I.	Open Life Sci. 2023 Jul 6;18(1):20220611. doi: 10.1515/biol-2022-0611. eCollection 2023.	Parashar A	Open Life Sci	2023	2023/07/10	PMC10329279		10.1515/biol-2022-0611
34101071	Automatic knee cartilage and bone segmentation using multi-stage convolutional neural networks: data from the osteoarthritis initiative	Gatti AA, Maly MR.	MAGMA. 2021 Dec;34(6):859-875. doi: 10.1007/s10334-021-00934-z. Epub 2021 Jun 8.	Gatti AA	MAGMA	2021	2021/06/08			10.1007/s10334-021-00934-z
32715584	Deep Learning Approach for Anterior Cruciate Ligament Lesion Detection: Evaluation of Diagnostic Performance Using Arthroscopy as the Reference Standard	Zhang L, Li M, Zhou Y, Lu G, Zhou Q.	J Magn Reson Imaging. 2020 Dec;52(6):1745-1752. doi: 10.1002/jmri.27266. Epub 2020 Jul 26.	Zhang L	J Magn Reson Imaging	2020	2020/07/28			10.1002/jmri.27266
35107593	Automated segmentation of magnetic resonance bone marrow signal: a feasibility study	von Brandis E, Jenssen HB, Avenarius DFM, Bjørnerud A, Flatø B, Tomterstad AH, Lilleby V, Rosendahl K, Sakinis T, Zadig PKK, Müller LO.	Pediatr Radiol. 2022 May;52(6):1104-1114. doi: 10.1007/s00247-021-05270-x. Epub 2022 Feb 2.	von Brandis E	Pediatr Radiol	2022	2022/02/02	PMC9107442		10.1007/s00247-021-05270-x
36292003	Anterior Cruciate Ligament Tear Detection Based on Deep Convolutional Neural Network	Joshi K, Suganthi K.	Diagnostics (Basel). 2022 Sep 26;12(10):2314. doi: 10.3390/diagnostics12102314.	Joshi K	Diagnostics (Basel)	2022	2022/10/27	PMC9600338		10.3390/diagnostics12102314
30040634	Deep Generative Adversarial Neural Networks for Compressive Sensing MRI	Mardani M, Gong E, Cheng JY, Vasanawala SS, Zaharchuk G, Xing L, Pauly JM.	IEEE Trans Med Imaging. 2019 Jan;38(1):167-179. doi: 10.1109/TMI.2018.2858752. Epub 2018 Jul 23.	Mardani M	IEEE Trans Med Imaging	2019	2018/07/25	PMC6542360	NIHMS1517794	10.1109/TMI.2018.2858752
23887984	Difficulty with learning of exercise instructions associated with 'working memory' dysfunction and frontal glucose hypometabolism in a patient with very mild subcortical vascular dementia with knee osteoarthritis	Takeda K, Meguro K, Tanaka N, Nakatsuka M.	BMJ Case Rep. 2013 Jul 25;2013:bcr2013008577. doi: 10.1136/bcr-2013-008577.	Takeda K	BMJ Case Rep	2013	2013/07/27	PMC3736114		10.1136/bcr-2013-008577
35637451	Development of convolutional neural network model for diagnosing meniscus tear using magnetic resonance image	Shin H, Choi GS, Shon OJ, Kim GB, Chang MC.	BMC Musculoskelet Disord. 2022 May 30;23(1):510. doi: 10.1186/s12891-022-05468-6.	Shin H	BMC Musculoskelet Disord	2022	2022/05/31	PMC9150332		10.1186/s12891-022-05468-6
34110037	Systematic evaluation of iterative deep neural networks for fast parallel MRI reconstruction with sensitivity-weighted coil combination	Hammernik K, Schlemper J, Qin C, Duan J, Summers RM, Rueckert D.	Magn Reson Med. 2021 Oct;86(4):1859-1872. doi: 10.1002/mrm.28827. Epub 2021 Jun 10.	Hammernik K	Magn Reson Med	2021	2021/06/10			10.1002/mrm.28827
35648374	Automatic Detection of Meniscus Tears Using Backbone Convolutional Neural Networks on Knee MRI	Hung TNK, Vy VPT, Tri NM, Hoang LN, Tuan LV, Ho QT, Le NQK, Kang JH.	J Magn Reson Imaging. 2023 Mar;57(3):740-749. doi: 10.1002/jmri.28284. Epub 2022 Jun 1.	Hung TNK	J Magn Reson Imaging	2023	2022/06/01			10.1002/jmri.28284
32614100	Self-supervised learning of physics-guided reconstruction neural networks without fully sampled reference data	Yaman B, Hosseini SAH, Moeller S, Ellermann J, Uğurbil K, Akçakaya M.	Magn Reson Med. 2020 Dec;84(6):3172-3191. doi: 10.1002/mrm.28378. Epub 2020 Jul 2.	Yaman B	Magn Reson Med	2020	2020/07/03	PMC7811359	NIHMS1661354	10.1002/mrm.28378
36067702	A cascade of preconditioned conjugate gradient networks for accelerated magnetic resonance imaging	Kim M, Chung W.	Comput Methods Programs Biomed. 2022 Oct;225:107090. doi: 10.1016/j.cmpb.2022.107090. Epub 2022 Aug 29.	Kim M	Comput Methods Programs Biomed	2022	2022/09/06			10.1016/j.cmpb.2022.107090
31166049	SANTIS: Sampling-Augmented Neural neTwork with Incoherent Structure for MR image reconstruction	Liu F, Samsonov A, Chen L, Kijowski R, Feng L.	Magn Reson Med. 2019 Nov;82(5):1890-1904. doi: 10.1002/mrm.27827. Epub 2019 Jun 5.	Liu F	Magn Reson Med	2019	2019/06/06	PMC6660404	NIHMS1028480	10.1002/mrm.27827
32755384	Diagnostic Accuracy of Quantitative Multicontrast 5-Minute Knee MRI Using Prospective Artificial Intelligence Image Quality Enhancement	Chaudhari AS, Grissom MJ, Fang Z, Sveinsson B, Lee JH, Gold GE, Hargreaves BA, Stevens KJ.	AJR Am J Roentgenol. 2021 Jun;216(6):1614-1625. doi: 10.2214/AJR.20.24172. Epub 2020 Aug 5.	Chaudhari AS	AJR Am J Roentgenol	2021	2020/08/07	PMC8862596	NIHMS1773740	10.2214/AJR.20.24172
35041598	NC-PDNet: A Density-Compensated Unrolled Network for 2D and 3D Non-Cartesian MRI Reconstruction	Ramzi Z, G R C, Starck JL, Ciuciu P.	IEEE Trans Med Imaging. 2022 Jul;41(7):1625-1638. doi: 10.1109/TMI.2022.3144619. Epub 2022 Jun 30.	Ramzi Z	IEEE Trans Med Imaging	2022	2022/01/18			10.1109/TMI.2022.3144619
35551485	Design and validation of a semi-automatic bone segmentation algorithm from MRI to improve research efficiency	Heckelman LN, Soher BJ, Spritzer CE, Lewis BD, DeFrate LE.	Sci Rep. 2022 May 12;12(1):7825. doi: 10.1038/s41598-022-11785-6.	Heckelman LN	Sci Rep	2022	2022/05/13	PMC9098419		10.1038/s41598-022-11785-6
31297614	Age Prediction Based on Brain MRI Image: A Survey	Sajedi H, Pardakhti N.	J Med Syst. 2019 Jul 11;43(8):279. doi: 10.1007/s10916-019-1401-7.	Sajedi H	J Med Syst	2019	2019/07/13			10.1007/s10916-019-1401-7
34968699	Evaluation on the generalization of a learned convolutional neural network for MRI reconstruction	Huang J, Wang S, Zhou G, Hu W, Yu G.	Magn Reson Imaging. 2022 Apr;87:38-46. doi: 10.1016/j.mri.2021.12.003. Epub 2021 Dec 27.	Huang J	Magn Reson Imaging	2022	2021/12/30			10.1016/j.mri.2021.12.003
30536427	SUSAN: segment unannotated image structure using adversarial network	Liu F.	Magn Reson Med. 2019 May;81(5):3330-3345. doi: 10.1002/mrm.27627. Epub 2018 Dec 10.	Liu F	Magn Reson Med	2019	2018/12/12	PMC7140982	NIHMS1572885	10.1002/mrm.27627
33075675	The optimisation of deep neural networks for segmenting multiple knee joint tissues from MRIs	Kessler DA, MacKay JW, Crowe VA, Henson FMD, Graves MJ, Gilbert FJ, Kaggie JD.	Comput Med Imaging Graph. 2020 Dec;86:101793. doi: 10.1016/j.compmedimag.2020.101793. Epub 2020 Sep 28.	Kessler DA	Comput Med Imaging Graph	2020	2020/10/19	PMC7721597		10.1016/j.compmedimag.2020.101793
33458865	A transfer learning approach for automatic segmentation of the surgically treated anterior cruciate ligament	Flannery SW, Kiapour AM, Edgar DJ, Murray MM, Beveridge JE, Fleming BC.	J Orthop Res. 2022 Jan;40(1):277-284. doi: 10.1002/jor.24984. Epub 2021 Jan 27.	Flannery SW	J Orthop Res	2022	2021/01/18	PMC8285460	NIHMS1664227	10.1002/jor.24984
34148167	MR-contrast-aware image-to-image translations with generative adversarial networks	Denck J, Guehring J, Maier A, Rothgang E.	Int J Comput Assist Radiol Surg. 2021 Dec;16(12):2069-2078. doi: 10.1007/s11548-021-02433-x. Epub 2021 Jun 20.	Denck J	Int J Comput Assist Radiol Surg	2021	2021/06/20	PMC8616894		10.1007/s11548-021-02433-x
37492386	uRP: An integrated research platform for one-stop analysis of medical images	Wu J, Xia Y, Wang X, Wei Y, Liu A, Innanje A, Zheng M, Chen L, Shi J, Wang L, Zhan Y, Zhou XS, Xue Z, Shi F, Shen D.	Front Radiol. 2023 Apr 18;3:1153784. doi: 10.3389/fradi.2023.1153784. eCollection 2023.	Wu J	Front Radiol	2023	2023/07/26	PMC10365282		10.3389/fradi.2023.1153784
36650496	Automatic segmentation of human knee anatomy by a convolutional neural network applying a 3D MRI protocol	Kulseng CPS, Nainamalai V, Grøvik E, Geitung JT, Årøen A, Gjesdal KI.	BMC Musculoskelet Disord. 2023 Jan 18;24(1):41. doi: 10.1186/s12891-023-06153-y.	Kulseng CPS	BMC Musculoskelet Disord	2023	2023/01/17	PMC9847207		10.1186/s12891-023-06153-y
33154515	Rapid mono and biexponential 3D-T(1ρ) mapping of knee cartilage using variational networks	Zibetti MVW, Johnson PM, Sharafi A, Hammernik K, Knoll F, Regatte RR.	Sci Rep. 2020 Nov 5;10(1):19144. doi: 10.1038/s41598-020-76126-x.	Zibetti MVW	Sci Rep	2020	2020/11/06	PMC7645759		10.1038/s41598-020-76126-x
36085911	DVS-Net: Dual-domain Variable Splitting Network for Accelerated Parallel MRI Data	Ding R, Bartlett J, Duan J, Duan Y.	Annu Int Conf IEEE Eng Med Biol Soc. 2022 Jul;2022:2219-2223. doi: 10.1109/EMBC48229.2022.9871425.	Ding R	Annu Int Conf IEEE Eng Med Biol Soc	2022	2022/09/10			10.1109/EMBC48229.2022.9871425
32882339	A multi-scale residual network for accelerated radial MR parameter mapping	Fu Z, Mandava S, Keerthivasan MB, Li Z, Johnson K, Martin DR, Altbach MI, Bilgin A.	Magn Reson Imaging. 2020 Nov;73:152-162. doi: 10.1016/j.mri.2020.08.013. Epub 2020 Sep 1.	Fu Z	Magn Reson Imaging	2020	2020/09/04	PMC7580302	NIHMS1626997	10.1016/j.mri.2020.08.013
29526784	Knee menisci segmentation using convolutional neural networks: data from the Osteoarthritis Initiative	Tack A, Mukhopadhyay A, Zachow S.	Osteoarthritis Cartilage. 2018 May;26(5):680-688. doi: 10.1016/j.joca.2018.02.907. Epub 2018 Mar 9.	Tack A	Osteoarthritis Cartilage	2018	2018/03/13			10.1016/j.joca.2018.02.907
35705930	Automated detection of anterior cruciate ligament tears using a deep convolutional neural network	Minamoto Y, Akagi R, Maki S, Shiko Y, Tozawa R, Kimura S, Yamaguchi S, Kawasaki Y, Ohtori S, Sasho T.	BMC Musculoskelet Disord. 2022 Jun 15;23(1):577. doi: 10.1186/s12891-022-05524-1.	Minamoto Y	BMC Musculoskelet Disord	2022	2022/06/15	PMC9199233		10.1186/s12891-022-05524-1
36573479	Democratization of deep learning for segmenting cartilage from MRIs of human knees: Application to data from the osteoarthritis initiative	Rodriguez-Vila B, Gonzalez-Hospital V, Puertas E, Beunza JJ, Pierce DM.	J Orthop Res. 2023 Aug;41(8):1754-1766. doi: 10.1002/jor.25509. Epub 2023 Jan 7.	Rodriguez-Vila B	J Orthop Res	2023	2022/12/27			10.1002/jor.25509
34753963	Uncovering associations between data-driven learned qMRI biomarkers and chronic pain	Morales AG, Lee JJ, Caliva F, Iriondo C, Liu F, Majumdar S, Pedoia V.	Sci Rep. 2021 Nov 9;11(1):21989. doi: 10.1038/s41598-021-01111-x.	Morales AG	Sci Rep	2021	2021/11/10	PMC8578418		10.1038/s41598-021-01111-x
30860285	MANTIS: Model-Augmented Neural neTwork with Incoherent k-space Sampling for efficient MR parameter mapping	Liu F, Feng L, Kijowski R.	Magn Reson Med. 2019 Jul;82(1):174-188. doi: 10.1002/mrm.27707. Epub 2019 Mar 12.	Liu F	Magn Reson Med	2019	2019/03/13	PMC7144418	NIHMS1566608	10.1002/mrm.27707
35494819	Multi-level pooling encoder-decoder convolution neural network for MRI reconstruction	Karnjanapreechakorn S, Kusakunniran W, Siriapisith T, Saiviroonporn P.	PeerJ Comput Sci. 2022 Mar 30;8:e934. doi: 10.7717/peerj-cs.934. eCollection 2022.	Karnjanapreechakorn S	PeerJ Comput Sci	2022	2022/05/02	PMC9044365		10.7717/peerj-cs.934
29584598	Use of 2D U-Net Convolutional Neural Networks for Automated Cartilage and Meniscus Segmentation of Knee MR Imaging Data to Determine Relaxometry and Morphometry	Norman B, Pedoia V, Majumdar S.	Radiology. 2018 Jul;288(1):177-185. doi: 10.1148/radiol.2018172322. Epub 2018 Mar 27.	Norman B	Radiology	2018	2018/03/28	PMC6013406	NIHMS967812	10.1148/radiol.2018172322
32691905	T(2) analysis of the entire osteoarthritis initiative dataset	Razmjoo A, Caliva F, Lee J, Liu F, Joseph GB, Link TM, Majumdar S, Pedoia V.	J Orthop Res. 2021 Jan;39(1):74-85. doi: 10.1002/jor.24811. Epub 2020 Jul 27.	Razmjoo A	J Orthop Res	2021	2020/07/22			10.1002/jor.24811
33966462	Which GAN? A comparative study of generative adversarial network-based fast MRI reconstruction	Lv J, Zhu J, Yang G.	Philos Trans A Math Phys Eng Sci. 2021 Jun 28;379(2200):20200203. doi: 10.1098/rsta.2020.0203. Epub 2021 May 10.	Lv J	Philos Trans A Math Phys Eng Sci	2021	2021/05/10			10.1098/rsta.2020.0203
36423533	Denoising of three-dimensional fast spin echo magnetic resonance images of knee joints using spatial-variant noise-relevant residual learning of convolution neural network	Zhao S, Cahill DG, Li S, Xiao F, Blu T, Griffith JF, Chen W.	Comput Biol Med. 2022 Dec;151(Pt A):106295. doi: 10.1016/j.compbiomed.2022.106295. Epub 2022 Nov 9.	Zhao S	Comput Biol Med	2022	2022/11/24			10.1016/j.compbiomed.2022.106295
35508147	Assessment of data consistency through cascades of independently recurrent inference machines for fast and robust accelerated MRI reconstruction	Karkalousos D, Noteboom S, Hulst HE, Vos FM, Caan MWA.	Phys Med Biol. 2022 Jun 8;67(12). doi: 10.1088/1361-6560/ac6cc2.	Karkalousos D	Phys Med Biol	2022	2022/05/04			10.1088/1361-6560/ac6cc2
34834515	Improved Deep Convolutional Neural Network to Classify Osteoarthritis from Anterior Cruciate Ligament Tear Using Magnetic Resonance Imaging	Awan MJ, Rahim MSM, Salim N, Rehman A, Nobanee H, Shabir H.	J Pers Med. 2021 Nov 9;11(11):1163. doi: 10.3390/jpm11111163.	Awan MJ	J Pers Med	2021	2021/11/27	PMC8617867		10.3390/jpm11111163
36603439	SwinGAN: A dual-domain Swin Transformer-based generative adversarial network for MRI reconstruction	Zhao X, Yang T, Li B, Zhang X.	Comput Biol Med. 2023 Feb;153:106513. doi: 10.1016/j.compbiomed.2022.106513. Epub 2022 Dec 31.	Zhao X	Comput Biol Med	2023	2023/01/05			10.1016/j.compbiomed.2022.106513
30306701	3D convolutional neural networks for detection and severity staging of meniscus and PFJ cartilage morphological degenerative changes in osteoarthritis and anterior cruciate ligament subjects	Pedoia V, Norman B, Mehany SN, Bucknor MD, Link TM, Majumdar S.	J Magn Reson Imaging. 2019 Feb;49(2):400-410. doi: 10.1002/jmri.26246. Epub 2018 Oct 10.	Pedoia V	J Magn Reson Imaging	2019	2018/10/12	PMC6521715	NIHMS1012205	10.1002/jmri.26246
34031789	A Coarse-to-Fine Framework for Automated Knee Bone and Cartilage Segmentation Data from the Osteoarthritis Initiative	Deng Y, You L, Wang Y, Zhou X.	J Digit Imaging. 2021 Aug;34(4):833-840. doi: 10.1007/s10278-021-00464-z. Epub 2021 May 24.	Deng Y	J Digit Imaging	2021	2021/05/25	PMC8455760		10.1007/s10278-021-00464-z
32523326	Networks for Joint Affine and Non-parametric Image Registration	Shen Z, Han X, Xu Z, Niethammer M.	Proc IEEE Comput Soc Conf Comput Vis Pattern Recognit. 2019 Jun;2019:4219-4228. doi: 10.1109/cvpr.2019.00435. Epub 2020 Jan 9.	Shen Z	Proc IEEE Comput Soc Conf Comput Vis Pattern Recognit	2019	2020/06/12	PMC7286599	NIHMS1033312	10.1109/cvpr.2019.00435
34460769	Enhanced Magnetic Resonance Image Synthesis with Contrast-Aware Generative Adversarial Networks	Denck J, Guehring J, Maier A, Rothgang E.	J Imaging. 2021 Aug 4;7(8):133. doi: 10.3390/jimaging7080133.	Denck J	J Imaging	2021	2021/08/30	PMC8404922		10.3390/jimaging7080133
36384059	A novel multi-atlas segmentation approach under the semi-supervised learning framework: Application to knee cartilage segmentation	Chadoulos CG, Tsaopoulos DE, Moustakidis S, Tsakiridis NL, Theocharis JB.	Comput Methods Programs Biomed. 2022 Dec;227:107208. doi: 10.1016/j.cmpb.2022.107208. Epub 2022 Oct 28.	Chadoulos CG	Comput Methods Programs Biomed	2022	2022/11/17			10.1016/j.cmpb.2022.107208
35339616	DBGAN: A dual-branch generative adversarial network for undersampled MRI reconstruction	Liu X, Du H, Xu J, Qiu B.	Magn Reson Imaging. 2022 Jun;89:77-91. doi: 10.1016/j.mri.2022.03.003. Epub 2022 Mar 24.	Liu X	Magn Reson Imaging	2022	2022/03/27			10.1016/j.mri.2022.03.003
33018271	High-Fidelity Accelerated MRI Reconstruction by Scan-Specific Fine-Tuning of Physics-Based Neural Networks	Hossein Hosseini SA, Yaman B, Moeller S, Akcakaya M.	Annu Int Conf IEEE Eng Med Biol Soc. 2020 Jul;2020:1481-1484. doi: 10.1109/EMBC44109.2020.9176241.	Hossein Hosseini SA	Annu Int Conf IEEE Eng Med Biol Soc	2020	2020/10/06	PMC8597413	NIHMS1753915	10.1109/EMBC44109.2020.9176241
34823835	Automated tibiofemoral joint segmentation based on deeply supervised 2D-3D ensemble U-Net: Data from the Osteoarthritis Initiative	Latif MHA, Faye I.	Artif Intell Med. 2021 Dec;122:102213. doi: 10.1016/j.artmed.2021.102213. Epub 2021 Nov 14.	Latif MHA	Artif Intell Med	2021	2021/11/26			10.1016/j.artmed.2021.102213
34988758	Entropy and distance maps-guided segmentation of articular cartilage: data from the Osteoarthritis Initiative	Li Z, Chen K, Liu P, Chen X, Zheng G.	Int J Comput Assist Radiol Surg. 2022 Mar;17(3):553-560. doi: 10.1007/s11548-021-02555-2. Epub 2022 Jan 6.	Li Z	Int J Comput Assist Radiol Surg	2022	2022/01/06			10.1007/s11548-021-02555-2
31402520	Fully automated patellofemoral MRI segmentation using holistically nested networks: Implications for evaluating patellofemoral osteoarthritis, pain, injury, pathology, and adolescent development	Cheng R, Alexandridi NA, Smith RM, Shen A, Gandler W, McCreedy E, McAuliffe MJ, Sheehan FT.	Magn Reson Med. 2020 Jan;83(1):139-153. doi: 10.1002/mrm.27920. Epub 2019 Aug 11.	Cheng R	Magn Reson Med	2020	2019/08/13	PMC6778709	NIHMS1041450	10.1002/mrm.27920
37426618	Evaluation of effects of small-incision approach treatment on proximal tibia fracture by deep learning algorithm-based magnetic resonance imaging	Li X, Yu H, Li F, He Y, Xu L, Xiao J.	Open Life Sci. 2023 Jul 6;18(1):20220624. doi: 10.1515/biol-2022-0624. eCollection 2023.	Li X	Open Life Sci	2023	2023/07/10	PMC10329276		10.1515/biol-2022-0624
32956805	Deriving new soft tissue contrasts from conventional MR images using deep learning	Wu Y, Li D, Xing L, Gold G.	Magn Reson Imaging. 2020 Dec;74:121-127. doi: 10.1016/j.mri.2020.09.014. Epub 2020 Sep 19.	Wu Y	Magn Reson Imaging	2020	2020/09/21	PMC7669656	NIHMS1632926	10.1016/j.mri.2020.09.014
31049802	Extending pretrained segmentation networks with additional anatomical structures	Ozdemir F, Goksel O.	Int J Comput Assist Radiol Surg. 2019 Jul;14(7):1187-1195. doi: 10.1007/s11548-019-01984-4. Epub 2019 May 2.	Ozdemir F	Int J Comput Assist Radiol Surg	2019	2019/05/04			10.1007/s11548-019-01984-4
35951046	Diabetes-associated thigh muscle degeneration mediates knee osteoarthritis-related outcomes: results from a longitudinal cohort study	Mohajer B, Moradi K, Guermazi A, Dolatshahi M, Zikria B, Najafzadeh N, Kalyani RR, Roemer FW, Berenbaum F, Demehri S.	Eur Radiol. 2023 Jan;33(1):595-605. doi: 10.1007/s00330-022-09035-4. Epub 2022 Aug 11.	Mohajer B	Eur Radiol	2023	2022/08/11	PMC10448875	NIHMS1916796	10.1007/s00330-022-09035-4
34876922	Complexity Assessment of Chronic Pain in Elderly Knee Osteoarthritis Based on Neuroimaging Recognition Techniques	Wu X, Liu J, Liu M, Wu T.	Comput Math Methods Med. 2021 Nov 28;2021:7344102. doi: 10.1155/2021/7344102. eCollection 2021.	Wu X	Comput Math Methods Med	2021	2021/12/08	PMC8645396		10.1155/2021/7344102
35364383	Improved-Mask R-CNN: Towards an accurate generic MSK MRI instance segmentation platform (data from the Osteoarthritis Initiative)	Felfeliyan B, Hareendranathan A, Kuntze G, Jaremko JL, Ronsky JL.	Comput Med Imaging Graph. 2022 Apr;97:102056. doi: 10.1016/j.compmedimag.2022.102056. Epub 2022 Mar 19.	Felfeliyan B	Comput Med Imaging Graph	2022	2022/04/01			10.1016/j.compmedimag.2022.102056
34617020	Synthesizing Quantitative T2 Maps in Right Lateral Knee Femoral Condyles from Multicontrast Anatomic Data with a Conditional Generative Adversarial Network	Sveinsson B, Chaudhari AS, Zhu B, Koonjoo N, Torriani M, Gold GE, Rosen MS.	Radiol Artif Intell. 2021 May 26;3(5):e200122. doi: 10.1148/ryai.2021200122. eCollection 2021 Sep.	Sveinsson B	Radiol Artif Intell	2021	2021/10/07	PMC8489449		10.1148/ryai.2021200122
33241856	Automated magnetic resonance image segmentation of the anterior cruciate ligament	Flannery SW, Kiapour AM, Edgar DJ, Murray MM, Fleming BC.	J Orthop Res. 2021 Apr;39(4):831-840. doi: 10.1002/jor.24926. Epub 2020 Dec 7.	Flannery SW	J Orthop Res	2021	2020/11/26	PMC8005419	NIHMS1651243	10.1002/jor.24926
35245407	Personalized Risk Model and Leveraging of Magnetic Resonance Imaging-Based Structural Phenotypes and Clinical Factors to Predict Incidence of Radiographic Osteoarthritis	Lee JJ, Namiri NK, Astuto B, Link TM, Majumdar S, Pedoia V.	Arthritis Care Res (Hoboken). 2023 Mar;75(3):501-508. doi: 10.1002/acr.24877. Epub 2022 Dec 10.	Lee JJ	Arthritis Care Res (Hoboken)	2023	2022/03/04			10.1002/acr.24877
32479671	Deep-learned short tau inversion recovery imaging using multi-contrast MR images	Kim S, Jang H, Jang J, Lee YH, Hwang D.	Magn Reson Med. 2020 Dec;84(6):2994-3008. doi: 10.1002/mrm.28327. Epub 2020 Jun 1.	Kim S	Magn Reson Med	2020	2020/06/02			10.1002/mrm.28327
33337584	Detection of Differences in Longitudinal Cartilage Thickness Loss Using a Deep-Learning Automated Segmentation Algorithm: Data From the Foundation for the National Institutes of Health Biomarkers Study of the Osteoarthritis Initiative	Eckstein F, Chaudhari AS, Fuerst D, Gaisberger M, Kemnitz J, Baumgartner CF, Konukoglu E, Hunter DJ, Wirth W.	Arthritis Care Res (Hoboken). 2022 Jun;74(6):929-936. doi: 10.1002/acr.24539. Epub 2022 Apr 1.	Eckstein F	Arthritis Care Res (Hoboken)	2022	2020/12/18	PMC9321555		10.1002/acr.24539
37292137	Automated MRI quantification of volumetric per-muscle fat fractions in the proximal leg of patients with muscular dystrophies	Huysmans L, De Wel B, Claeys KG, Maes F.	Front Neurol. 2023 May 24;14:1200727. doi: 10.3389/fneur.2023.1200727. eCollection 2023.	Huysmans L	Front Neurol	2023	2023/06/09	PMC10244517		10.3389/fneur.2023.1200727
33747334	Dense Recurrent Neural Networks for Accelerated MRI: History-Cognizant Unrolling of Optimization Algorithms	Hosseini SAH, Yaman B, Moeller S, Hong M, Akçakaya M.	IEEE J Sel Top Signal Process. 2020 Oct;14(6):1280-1291. doi: 10.1109/jstsp.2020.3003170. Epub 2020 Jun 17.	Hosseini SAH	IEEE J Sel Top Signal Process	2020	2021/03/22	PMC7978039	NIHMS1632252	10.1109/jstsp.2020.3003170
36581003	A More Posterior Tibial Tubercle (Decreased Sagittal Tibial Tubercle-Trochlear Groove Distance) Is Significantly Associated With Patellofemoral Joint Degenerative Cartilage Change: A Deep Learning Analysis	Namiri NK, Càliva F, Martinez AM, Pedoia V, Lansdown DA.	Arthroscopy. 2023 Jun;39(6):1493-1501.e2. doi: 10.1016/j.arthro.2022.11.040. Epub 2022 Dec 26.	Namiri NK	Arthroscopy	2023	2022/12/29			10.1016/j.arthro.2022.11.040
34359308	Comparison of the Predicting Performance for Fate of Medial Meniscus Posterior Root Tear Based on Treatment Strategies: A Comparison between Logistic Regression, Gradient Boosting, and CNN Algorithms	Lee JI, Kim DH, Yoo HJ, Choi HG, Lee YS.	Diagnostics (Basel). 2021 Jul 7;11(7):1225. doi: 10.3390/diagnostics11071225.	Lee JI	Diagnostics (Basel)	2021	2021/08/07	PMC8304966		10.3390/diagnostics11071225
31644542	Optimized fast GPU implementation of robust artificial-neural-networks for k-space interpolation (RAKI) reconstruction	Zhang C, Hosseini SAH, Weingärtner S, Uǧurbil K, Moeller S, Akçakaya M.	PLoS One. 2019 Oct 23;14(10):e0223315. doi: 10.1371/journal.pone.0223315. eCollection 2019.	Zhang C	PLoS One	2019	2019/10/24	PMC6808331		10.1371/journal.pone.0223315
30903682	Quantification of patellofemoral cartilage deformation and contact area changes in response to static loading via high-resolution MRI with prospective motion correction	Lange T, Taghizadeh E, Knowles BR, Südkamp NP, Zaitsev M, Meine H, Izadpanah K.	J Magn Reson Imaging. 2019 Nov;50(5):1561-1570. doi: 10.1002/jmri.26724. Epub 2019 Mar 23.	Lange T	J Magn Reson Imaging	2019	2019/03/24			10.1002/jmri.26724
33585029	Studying human-AI collaboration protocols: the case of the Kasparov's law in radiological double reading	Cabitza F, Campagner A, Sconfienza LM.	Health Inf Sci Syst. 2021 Feb 5;9(1):8. doi: 10.1007/s13755-021-00138-8. eCollection 2021 Dec.	Cabitza F	Health Inf Sci Syst	2021	2021/02/15	PMC7864624		10.1007/s13755-021-00138-8
33640877	[Segmentation of knee cartilages in MR images with artificial intelligence]	Szoldán P, Egyed Z, Szabó E, Somogyi J, Hangody G, Hangody L.	Orv Hetil. 2021 Feb 28;162(9):352-360. doi: 10.1556/650.2021.32034.	Szoldán P	Orv Hetil	2021	2021/02/28			10.1556/650.2021.32034
36343061	Development of convolutional neural network model for diagnosing tear of anterior cruciate ligament using only one knee magnetic resonance image	Shin H, Choi GS, Chang MC.	Medicine (Baltimore). 2022 Nov 4;101(44):e31510. doi: 10.1097/MD.0000000000031510.	Shin H	Medicine (Baltimore)	2022	2022/11/07	PMC9646554		10.1097/MD.0000000000031510
34524564	Automatic segmentation of whole-body adipose tissue from magnetic resonance fat fraction images based on machine learning	Wang Z, Cheng C, Peng H, Qi Y, Wan Q, Zhou H, Qu S, Liang D, Liu X, Zheng H, Zou C.	MAGMA. 2022 Apr;35(2):193-203. doi: 10.1007/s10334-021-00958-5. Epub 2021 Sep 15.	Wang Z	MAGMA	2022	2021/09/15			10.1007/s10334-021-00958-5
